# Boosting Output Performance of Triboelectric Nanogenerator via Interface Self-Regulation Strategy

**DOI:** 10.34133/research.0906

**Published:** 2025-09-25

**Authors:** Yanrui Zhao, Yuming Feng, Qi Gao, Hengyu Li, Xin Guo, Jianlong Wang, Xinxian Wang, Lu Dong, Yang Yu, Zhong Lin Wang, Tinghai Cheng

**Affiliations:** ^1^ Beijing Institute of Nanoenergy and Nanosystems, Chinese Academy of Sciences, Beijing 101400, China.; ^2^ School of Nanoscience and Engineering, University of Chinese Academy of Sciences, Beijing 100049, China.; ^3^ Guangzhou Institute of Blue Energy, Knowledge City, Huangpu District, Guangzhou 510555, China.

## Abstract

The long-term durability of triboelectric nanogenerators (TENGs) remains a critical challenge for their practical deployment. Although approaches like reducing interfacial friction or contact duration can enhance durability, they often compromise electrical performance. The charge self-excitation method can improve the output performance. However, when it is introduced into the sliding mode with small capacitance change, it increases the complexity of the circuit and cannot solve the problem of charge attenuation caused by material wear. Herein, we propose a self-regulation strategy that concurrently controls the interface contact state and contact force. This approach synergistically combines the advantages of both sliding and contact-separation configurations, enabling the triboelectric materials to micro-slide and deform adaptively, ensuring stable dynamic interfacial contact under minimal normal pressure. Such a mechanism promotes strong electron cloud overlap at the microscale, thereby enhancing charge transfer efficiency. Compared to conventional TENGs, the self-regulating TENG achieves a 72.5-fold reduction in frictional force and a 13-fold increase in energy output. Furthermore, a wireless self-powered sensing system is integrated, achieving a power density of 242.4 mW/m^2^ under real water flow conditions. The system maintains 97.6% of the initial output after 10 h of continuous operation, confirming the practical feasibility of the proposed approach. This work presents a universal method to enhance the electrical performance and durability of TENGs, paving the way for their broader application.

## Introduction

With the rapid development of artificial intelligence and 5G technology, the number of distributed sensors is continuously increasing. Traditional lithium batteries suffer from limited energy storage efficiency and environmental issues, restricting their use in powering distributed sensors. Developing novel environmental energy harvesting technologies offers promising solutions to these challenges [1–3]. Among the various energy-harvesting technologies, triboelectric nanogenerators (TENGs), based on the principles of triboelectrification and electrostatic induction [4–6], have garnered considerable attention in recent years due to their simple fabrication, low cost, wide material selection [7,8], and high power density [9–12]. Since their invention by Wang’s group in 2012 [13], TENGs have demonstrated the ability to effectively convert ambient high-entropy energy into electricity, showing great promise as a sustainable energy supply solution [14–16]. Despite having these advantages, TENGs suffer from charge transfer processes induced by interfacial friction, which inevitably cause material wear at the contact interfaces. This wear gradually degrades their output performance and long-term reliability, posing a critical challenge to practical implementation.

To address the above challenges [17], several methods have been proposed, including intermittent contact operation [18,19], noncontact mode [20], soft contact mode [21], rolling mode [22,23], and so on. Intermittent contact and noncontact modes can effectively minimize or even eliminate interface wear, markedly enhancing the durability of TENGs. However, when the interface separates, the surface charges dissipate, leading to a decrease in TENG’s output performance [24]. The soft contact approach reduces friction between the tribo-materials, achieving high durability, but at the cost of lower interface contact efficiency. The rolling mode is a simple and effective technique that transforms sliding friction into rolling friction by shaping friction materials into spherical or cylindrical forms, thereby markedly reducing material wear. Nevertheless, this point or line contact method results in a smaller contact area between the materials, which reduces their charge generation capacity. These methods offer effective means to enhance the durability of TENGs, but they often compromise the charge transfer efficiency of the triboelectric materials. In addition, the charge self-excitation method can enhance the output performance [25], but it increases the complexity of the circuit and still cannot solve the problem of material wear when introduced into the sliding mode [26]. Previous studies have shown that electron transfer occurs only when the interatomic distance between 2 materials is smaller than the typical bond length, placing it within the region of electron cloud overlap [27]. Reducing interfacial friction or shortening the contact time decreases electron cloud overlap, thereby weakening the electrification capability of the materials. Therefore, achieving a balance between reduced friction and adequate electron cloud overlap at the tribo-material interface is essential for realizing both enhanced durability and optimal electrical performance.

Herein, we present a self-regulation strategy for concurrently controlling the interface contact state and contact force, which enables the triboelectric materials to micro-slide and deform adaptively, ensuring stable dynamic interface contact under minimal normal pressure. This mechanism promotes strong electron cloud overlap at the microscale, thereby enhancing the output performance. In contrast to the fixed electrode contact mode TENG (CF-TENG), the self-regulating TENG (SR-TENG) exhibits a 72.5-fold reduction in frictional force and a 13-fold increase in output energy. Through the optimization of structural parameters and an ingenious multi-layer synchronous stacking design, SR-TENG achieves a further enhancement in output. Subsequently, we develop an integrated prototype based on SR-TENG for the continuous and stable harvesting of water flow energy. Finally, we establish a wireless self-powered sensing system to support water environment monitoring, confirming the feasibility of this strategy in practical applications. This study proposes a novel method for boosting electrical output performance and durability, promoting the application of TENGs in IoT.

## Results

### Proposal and implementation of interface self-regulation strategy

**Fig. 1.**
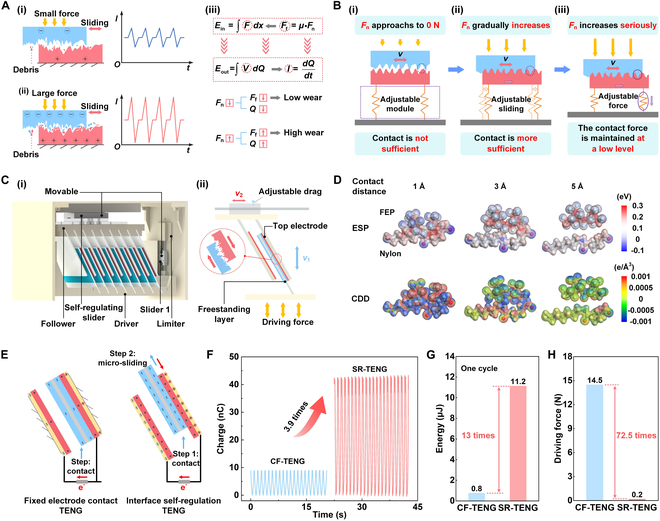
Implementation strategy and working mechanism of SR-TENG. (A) Output performance and wear of TENG as a function of contact force: small force (i), large force (ii), and their interrelationship (iii). (B) Conceptual diagram of implementation process of the interface self-regulation strategy: insufficient contact (i), sufficient contact (ii), and minimal contact force maintenance (iii). (C) Structure scheme (i) and working mechanism (ii) of SR-TENG. (D) Performance enhancement mechanism via DFT simulation calculation. (E) Configuration of CF-TENG and SR-TENG. (F) Output charge comparison of CF-TENG and SR-TENG. Comparison of (G) energy and (H) driving force between CF-TENG and SR-TENG.

The material interfaces of TENG invariably lead to wear issues during the triboelectrification process. When the contact force at the interfaces is relatively low, TENG’s output performance is small, and the wear is also minimal, as shown in Fig. 1A(i). When the contact force between the interfaces is very large, the contact of the friction surface is more compact, TENG has better electrical performance. But this gives rise to excessive wear, thereby influencing the durability of TENG, as illustrated in Fig. 1A(ii). This makes it difficult for TENG to simultaneously attain high durability and electrical performance. The phenomenon can be accounted for by the equation shown in Fig. 1A(iii) [28–32].

To address this issue, we put forward a self-regulation strategy for interface contact state and contact force. As depicted in Fig. 1B, this is the conceptual diagram of the implementation process. When the applied normal force *F*_n_ to TENG approaches 0 N, the contact between 2 material interfaces is inadequate, as depicted in Fig. 1B(i). As applied force *F*_n_ increases gradually, the adjustable module causes relative micro-sliding displacement and deformation of the triboelectric material. This results in closer dynamic contact and higher electrical performance, as illustrated in Fig. 1B(ii). When applied force *F*_n_ increases sharply, the adjustable module can absorb the excessive force, keeping the contact force between the 2 friction surfaces always at a relatively low level. Therefore, excessive wear is avoided during the operation of TENG, effectively enhancing its durability, as shown in Fig. 1B(iii). The demonstration animation of the interface self-regulation implementation concept is shown in Movie S1.

An interface SR-TENG is designed, as shown in Fig. 1C, based on the above strategy. SR-TENG mainly consists of a driving component, a driven component, and a power generation unit. The driving component encompasses a driver, slider 1, and a limiter. The driven component consists of a follower and self-regulating slider. The freestanding layer of the power generation unit and the 2 side electrodes are respectively connected to the driver and the follower via carbon fiber substrates, as depicted in Fig. 1C(i). It is notable that the implementation of this strategy encompasses the coordinated movement of both the electrodes and the freestanding layer. When exposed to the upward pressure and downward tension from the driver, the freestanding layer is capable of reciprocating vertically and undergoes alternating contact and micro-sliding motions with the electrodes on both sides. The self-regulating slider drives the electrodes on either side to be compelled to move to the right or left, thereby generating electrical energy. Slider 1 is employed to sustain the consistent linear motion of the freestanding layer, and the limiter is utilized to regulate its moving distance. The self-regulating slider is movable and possesses adjustable drag. On the one hand, it is utilized to maintain the linear motion of the electrode plates on both sides, thereby enabling the triboelectric materials to adaptively micro-slide and deform, and increasing the generation of charge at the contact interfaces. On the other hand, by setting its drag, a small normal pressure can be consistently maintained at the interfaces, enhancing TENG durability [Fig. 1C(ii)]. The working principle of SR-TENG is depicted in Fig. S1. The force analysis of SR-TENG during operational processes is shown in Fig. S2, and the equations of force analysis are delineated (Note S1). Initially, when the fluorinated ethylene propylene (FEP) film with fewer surface charges is close to the Nylon film surface, electrons transfer from the top electrode to bottom electrode through circuitry [Fig. S1A(i)]. Upon contact between the freestanding layer and top electrode, all electrons accumulate at the bottom electrode [Fig. S1A(ii)]. When the freestanding layer moves further upward, relative micro-sliding happens at the FEP and Nylon surfaces, increasing real tribo-materials contact region, thereby enhancing the surface charge generated [Fig. S1A(iii)]. After several cycles of operation, the charge accumulation gradually reaches saturation [Fig. S1A(iv)]. Subsequently, it enters the stable operation stage, as shown in Fig. S1B. During each operational cycle, the FEP film on the freestanding layer alternates with the Nylon film on both sides of the electrode to generate micro-slide to maintain the increased surface charge of the triboelectric material.

To deeply understand the microscopic mechanism of this performance enhancement, we calculate the interface electrostatic potential (ESP) and charge density differences (CDDs) at the molecular scale via density functional theory (DFT). As shown in Fig. S3, we quantitatively evaluate the actual contact area change caused by the micro-sliding of the material interface by adjusting the molecular distance of the FEP/Nylon interface model. The calculation results are shown in Fig. 1D. When the initial distance is large (5 Å), the interface ESP difference and CDD are both at a low level. As the molecular distance decreases (simulating the close contact caused by micro-sliding), the interface electron cloud overlap is substantially enhanced, which effectively promotes the charge transfer during the contact electrification process. When the distance is reduced to 1 Å, the system reaches the maximum ESP difference, and the CDD diagram also clearly shows the most pronounced charge transfer characteristics. In addition, according to the existing research results [33–35], the performance enhancement of SR-TENG proposed in this paper can be represented by the equation:

$$Q=\frac{2(\sigma +∆\sigma )\mathrm{Sx}}{d_{0}+g}$$ (1)

where *Q* is the transferred charge, *σ* is the initial charge density of the triboelectric layers, Δ*σ* is the enhanced charge density resulting from the self-regulation mechanism, *S* is the electrode surface area, *x* is the distance from the electrode to the dielectric layer, *d*_0_ is the effective thickness constant of dielectric material, and *g* is the distance between the 2 electrodes.

To evaluate the feasibility of the interface self-regulation strategy, the performances of SR-TENG and CF-TENG are compared (Fig. 1E). SR-TENG’s output charge increases by 3.9 times compared with CF-TENG (Fig. 1F). The output energy of SR-TENG increases by 13 times compared with CF-TENG, and the driving force decreases by approximately 72.5 times (Fig. 1G and H). The marked reduction in the frictional force at the materials interface indicates a marked decrease in wear.

### Output performance and parameter optimization of SR-TENG

**Fig. 2.**
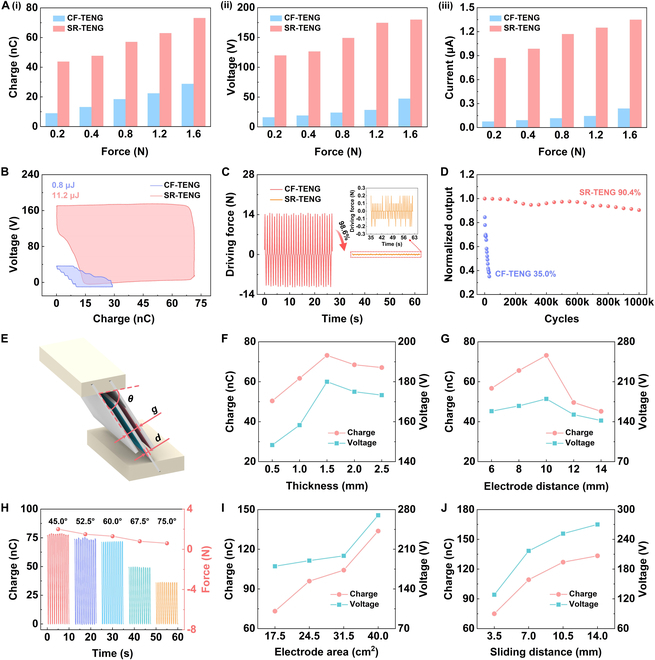
Results of increasing output performance and parameter optimization for SR-TENG. (A) Comparison of output charge (i), voltage (ii), and current (iii) between CF-TENG and SR-TENG under different interface contact forces. (B) Comparison of output energy between CF-TENG and SR-TENG. (C) Self-regulation effect of SR-TENG on driving force. (D) Comparison of durability between CF-TENG and SR-TENG. (E) Structural parameter diagram of single-layer SR-TENG. Dependence of SR-TENG output charge and voltage on (F) freestanding layer substrate thicknesses, (G) distances between 2 electrodes, and (H) layout angles. SR-TENG output charge and output voltage at various (I) electrode areas and (J) sliding distances.

To explore the output performance and mechanical characteristics of SR-TENG and to optimize its structural parameters, an experimental test system is built (Fig. S4). Note S2 illustrates the system’s detailed composition. The 2-dimensional (2D) diagram of size parameters of SR-TENG is shown in Fig. S5. Firstly, under different contact forces (shown as in Table S2) of the friction surfaces, the output charge, voltage, and current of both SR-TENG and CF-TENG are tested (Fig. 2A). Figures S6 and S7 demonstrate the electrical performance waveform diagrams of SR-TENG and CF-TENG. Under the same contact force, the performance of SR-TENG is markedly better than that of CF-TENG with a drive frequency of 1.0 Hz. Furthermore, with increasing contact force, the electrical performance of both devices improves, which is consistent with previous reports [36]. It should be noted that, under operational conditions of 0.2-N contact force and 27-mm stroke (*h*), SR-TENG exhibits an output charge of 43.8 nC, a voltage of 120 V, and a current of 0.87 μA. Compared with CF-TENG, these values have increased by approximately 3.9, 6.4, and 10.5 times, respectively. As demonstrated in Movie S2, SR-TENG exhibits a more excellent charge generation capability. Figure S8 reveals that the output power of SR-TENG and CF-TENG reaches 108.1 and 8.6 μW, respectively. Figure S9 presents output current of SR-TENG and CF-TENG at various load resistances. Figure 2B presents the comparison of output energy in one cycle. Output energy of SR-TENG demonstrates a 13-fold improvement over CF-TENG. In addition, the performance comparison experiments of different triboelectric material pairs prove the universality of SR-TENG in performance improvement (Fig. S10).

Subsequently, the driving forces required for SR-TENG and CF-TENG are quantified (Fig. 2C). At a stroke *h* of 27 mm, the driving force required by CF-TENG is 14.5 N, while that of SR-TENG is greatly reduced to only 0.2 N. According to the force analysis equation (Note S1), the frictional force of SR-TENG is decreased by 72.5 times compared with CF-TENG. This is attributed to the self-regulation function of SR-TENG. Specifically, the movable and low-drag characteristics of the self-regulating slider in SR-TENG facilitate the adaptive displacement and deformation of the triboelectric materials and result in a substantial reduction in frictional force. This also indicates that SR-TENG has high durability and can be readily activated with only a small driving force. Movie S3 shows the effect of SR-TENG in reducing the driving force. Then, the normalized output of transferred charge is compared for both SR-TENG and CF-TENG (Fig. 2D). SR-TENG maintains at 90.4% of the maximum value after 11 d (1,000,000 cycles), yet CF-TENG declines to 35.0% after 30,000 cycles. The electrical output of SR-TENG and CF-TENG during consecutive operations is shown in Fig. S11. In addition, the impact of different drag settings of self-regulating slider on the driving force required for SR-TENG is investigated. The experimental results demonstrate that the driving force ascends steadily with the increasing drag of self-regulating slider, which is largely consistent with the theoretical values (Fig. S12 and Table S2).

Further, the influences of the freestanding layer substrate thickness *d*, the interelectrode distance *g*, the electrode arrangement angle *θ*, the electrode area *S*, and the freestanding layer sliding distance *K* to output performance of SR-TENG are explored, at conditions where drag of self-regulating slider is 1.2 N, the stroke *h* is 27 mm, and the frequency is 1.0 Hz. The structure of single-layer SR-TENG is illustrated in Fig. 2E. By increasing the freestanding layer substrate thickness *d*, the output charge and output voltage show a tendency to firstly increase and then slowly reduce at an electrode distance *g* of 10 mm and an angle *θ* of 60.0°, as shown in Fig. 2F. The carbon fiber substrates with thicknesses *d* of 0.5 and 1.0 mm have relatively low stiffness, and there is obvious elastic deformation during the movement, resulting in insufficient contact and low electrical output. When the thickness is 1.5 mm, the output charge and output voltage reach the maximum values of 73.2 nC and 180 V, respectively. When the thickness is larger, due to the excessive stiffness of the carbon fiber substrate, it also leads to insufficient contact. With the distance *g* between the electrodes on both sides remaining unchanged, the larger the thickness *d* of the freestanding layer substrate, the shorter the charge transfer time. Therefore, the output current does not reduce as thickness of freestanding layer substrate is 2.0 and 2.5 mm, as shown in Fig. S13A. Maintaining thickness of freestanding layer substrate to 1.5 mm and arrangement angle to 60.0°, the output charge and output voltage exhibit a tendency to increase and then decrease as the distance between the electrodes on both sides increases. When the distance between the electrodes is 10 mm, the output performance reaches its maximum, as shown in Fig. 2G. Additionally, output current is gradually reduced as the electrode distance increases, as illustrated in Fig. S13B. This is because a larger distance results in a longer charge transfer time. When the thickness of the freestanding layer substrate is 1.5 mm and the electrode distance is 10 mm, the influence of the electrode arrangement angle *θ* upon output and driving force of SR-TENG is explored (Fig. 2H). As the angle *θ* increases, both output charge and driving force decrease, and output current also diminishes (Fig. S13C). The decreasing trend of the driving force is consistent with the force analysis results of SR-TENG (Table S3). When the angle *θ* is small, the driving force is high, indicating greater friction and wear on the material surface. Conversely, when the angle *θ* is 67.5° and 75.0°, the output performance is markedly reduced, affecting the practical application of SR-TENG. Therefore, the electrode arrangement angle *θ* of SR-TENG is set to 60.0°. Next, the effects of the increase in the electrode area *S* and the sliding distance *K* of the freestanding layer on SR-TENG’s electrical output are tested. The experimental results demonstrate that both the output charge and the output voltage exhibit a gradually increasing trend (Fig. 2I and J), and the output current shows a similar trend (Fig. S14A and B). Through parameter optimization, the output charge, output voltage, and output current reach 133.8 nC, 270 V, and 1.54 μA, respectively. These findings show that SR-TENG possesses durability, and its output performance has been enhanced through parameter optimization.

### Electrical properties of the multi-layer SR-TENG

**Fig. 3.**
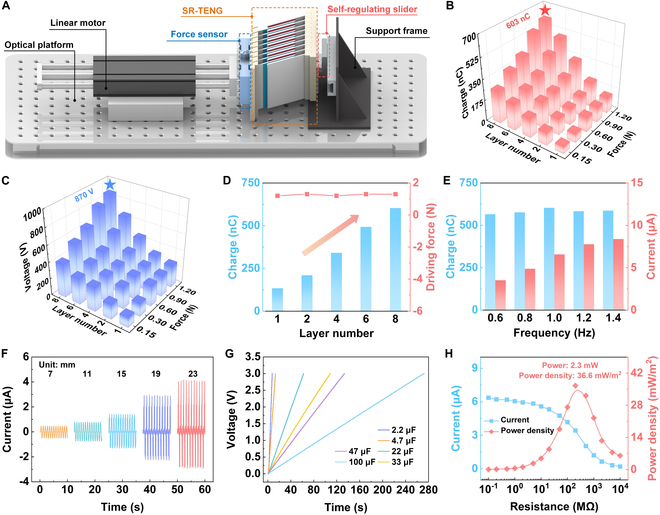
Multi-layer SR-TENG electrical output. (A) Experimental measurement platform schematic. The (B) charge and (C) voltage outputs of SR-TENG at various number of layers and slider drag. (D) Output charge and driving force of SR-TENG by different layer numbers at 1.2-N slider drag. (E) Output charge and output current of SR-TENG at variable excitation frequencies. (F) Multi-layer SR-TENG output current at varying strokes. (G) Charging curves of multi-layer SR-TENG for capacitors with different capacitances. (H) Multi-layer SR-TENG output power density under various external loads.

To further enhance the power generation performance, we design a multi-layer SR-TENG using a simple synchronous stacking method. The electrodes and freestanding layers of the multi-layer SR-TENG are designed with identical spacing and angles. Under the action of the self-regulating slider, the freestanding layers of all units undergo periodic synchronous contact-sliding motion with the electrodes on both sides. The flexible sponge on the substrate ensures full contact of the friction surfaces of all units during synchronous motion. The multi-layer SR-TENG is driven by a linear motor to systematically characterize its output performance under various operating conditions. As shown in Fig. 3A, the test setup includes a linear motor, a force sensor, SR-TENG, and a support frame. Due to the synchronous motion of the power generation units, there is no need to address the rectification issue of individual TENGs. This allows every TENG unit to become directly linked in parallel to output electrical energy.

The variation in charge and voltage outputs of SR-TENG as the layer number and slider drag increase when frequency is 1.0 Hz (Fig. 3B and C). The higher the slider drag, the greater the output charge and output voltage, for the same number of layers. For the same slider drag, the output performance increases steadily as the layer number rises. The maximum charge and voltage outputs of the multilayer SR-TENG reach 603 nC and 870 V, respectively, when the number of layers is 8 and the slider drag is 1.2 N. Additionally, the force sensor characterizes the driving force required by SR-TENG at different numbers of layers when the frequency is 1.0 Hz and the slider drag is 1.2 N (Fig. 3D). Markedly, as the layer number increases, the SR-TENG’s output charge rises steadily, while the driving force remains almost constant at approximately 1.3 N. This can be attributed to the unique self-regulating design of SR-TENG, which ensures that the required driving force is primarily governed by the slider drag and has little dependence on layer number. As the layers are increased, the slider drag is theoretically distributed evenly across each layer, but the total driving force to overcome the slider drag to operate multi-layer SR-TENG remains constant. This implies that SR-TENG can be designed with more layers as required, and by simply adjusting the slider drag, high output performance, high durability, and a driving force compatible with the input mechanical energy can be achieved.

We evaluate the influence of different external excitation conditions to electrical output of SR-TENG. Firstly, Fig. 3E demonstrates how driving frequency modulates SR-TENG’s electrical performance. Output current gradually increases with increasing frequency, while the output charge remains largely unchanged. This is because the increased frequency accelerates the charge transfer rate. When frequency is 1.4 Hz, the output charge is 587 nC and output current is 8.38 μA. Fig. S15 shows output voltages at different frequencies. Figure 3F depicts output performance of SR-TENG under different strokes. The output current steadily rises with increasing stroke. When the stroke exceeds 15 mm, the self-regulating slider of SR-TENG begins to take effect, causing relative sliding at the contact interfaces, leading to a noticeable increase in the output current. At a stroke of 23 mm, the output current reaches 4.14 μA. Notably, when the stroke is less than 15 mm, the freestanding layer of SR-TENG does not contact the electrode material interface but still maintains a certain output current. This is due to SR-TENG’s excellent noncontact working ability. At a frequency of 1.0 Hz, SR-TENG charges a 100-μF capacitor up to 3 V in 273 s, demonstrating its high energy harvesting efficiency under these conditions, as shown in Fig. 3G.

Finally, multi-layer SR-TENG peak current and peak power are measured with varying external loads at 1.0-Hz frequency. The curve indicates that the peak power is 2.3 mW, and the peak power density is 36.6 mW/m^2^, as shown in Fig. 3H.

### Output performance of the integrated prototype in water flow

**Fig. 4.**
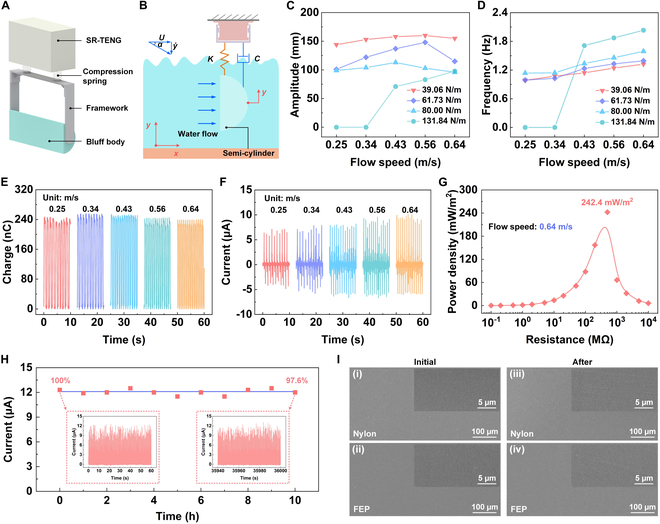
Electrical output evaluation under water flow conditions. (A) Schematic diagram of the integrated prototype. (B) Spring-damp system model of the designed prototype in water flow. (C) Amplitude of the bluff body with various spring stiffness. (D) Bluff body frequency at varying spring stiffness. Prototype (E) output charge and (F) current under various flow velocities. (G) Power density of the prototype at various external loads under 0.64 m/s flow velocity. (H) Prototype output current variation during continuous operation for 10 h in water flow. (I) SEM images of Nylon and FEP film before (i and ii) and after (iii and iv) durability testing.

In order to validate the feasibility of the presented approach for environmental energy collection, this paper designs a galloping-driven TENG (GDSR-TENG) for water flow energy collection (Fig. 4A). Figure S16 gives the photograph of GDSR-TENG device prototype. The main components of GDSR-TENG include SR-TENG, compression spring, framework, and bluff body. A water flow energy harvesting system is constructed to evaluate the output of GDSR-TENG under various conditions (Fig. 4B). Specifically, the semi-circular bluff body located underwater captures fluid energy, while the SR-TENG fixed above the water surface converts the reciprocating vibration energy of the bluff body to electrical energy. Notably, the independent design of the energy capture component and the power generation component of GDSR-TENG effectively isolates the influence of water on the generator, contributing to the sustainable operation of integrated prototype. The movement process of the GDSR-TENG device under the action of water flow is detailed (Fig. S17 and Movie S4).

The amplitude and frequency of the bluff body are systematically investigated at various flow speed under different spring stiffness coefficients (Fig. 4C and D). For small stiffness coefficients, the amplitude values of the bluff body are all large within the entire test flow velocity range, and the frequency will gradually increase with increasing fluid speed between 0.25 and 0.64 m/s. Compared to the stiffness coefficients of 39.06 and 80.00 N/m, the bluff body with a compression spring stiffness coefficient of 61.73 N/m achieves balanced amplitude and frequency within the entire test flow speed range. When the stiffness coefficient is too large, at 131.84 N/m, the frequency of the bluff body during operation is high but the amplitude is small. In addition, under 0.34 m/s flow velocity, both the amplitude and frequency of the bluff body are 0, indicating that the system requires a higher starting flow speed. Therefore, we choose a compression spring with a stiffness coefficient of 61.73 N/m as a component of GDSR-TENG and carry out its output performance test under water flow.

As the flow velocity is increased from 0.25 to 0.64 m/s, prototype output charge initially exhibits a gradual increase, followed by a gradual decrease, and finally stabilizes approximately 240 nC at 0.64 m/s (Fig. 4E). A comparable variation is observed in the output voltage (Fig. S18). As flow velocity increases, the output current gradually raises (Fig. 4F). This is because with increasing flow speed, the frequency of the bluff body increases, resulting in a corresponding growth of both output current and duty cycle. The output current is approximately 10 μA at 0.64 m/s flow velocity. Additionally, the peak power output of GDSR-TENG under varying load resistances is tested, and the power density is obtained using the size of the active area (Fig. 4G). At 0.64 m/s flow velocity, the prototype achieves a peak power density of 242.4 mW/m^2^ with a 500-MΩ load, which is better than the output performance of previously reported works on water flow energy harvesting (Fig. S19 and Table S1) [37–42]. The output currents under different load resistances are presented in Fig. S20.

To demonstrate the reliability of long-term power supply of GDSR-TENG in the service environment, its durability is tested (Fig. 4H). Following continuous operation in the water flow for 10 h, the GDSR-TENG current output retains 97.6% of the initial value, demonstrating robust output capability under prolonged humid conditions. The inset in Fig. 4H illustrates current waveforms at the beginning and the end of the test. Additionally, Fig. 4I presents a comparative scanning electron microscope (SEM) morphology analysis of Nylon and FEP film surfaces, contrasting unused (i and ii) with those following 10 h of continuous testing (iii and iv). It can be observed that only minor scratches appear at the interfaces after the test, confirming the stable output and high durability of GDSR-TENG in practical applications.

### Applications of the integrated prototype

**Fig. 5.**
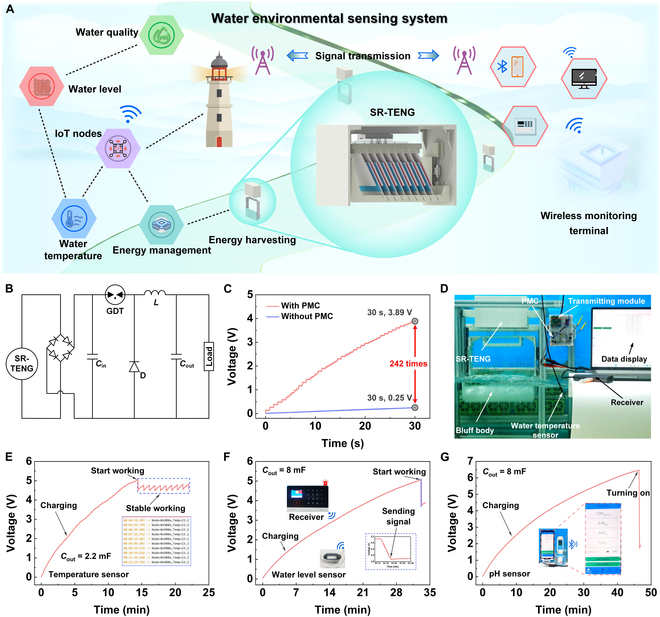
Application demonstration of the designed prototype. (A) Conceptual illustration of a wireless self-powered sensing system for water environment. (B) Circuit diagram of the prototype with a PMC. (C) Comparison of the capacitor charging speeds with and without the PMC. (D) Photo of the wireless self-powered sensing system. Voltage curves of (E) the wireless self-powered water temperature, (F) water level, and (G) water quality sensing process.

A wireless self-powered sensing system for a water environment is established to verify the feasibility of this suggested strategy for real-world deployment (Fig. 5A). The system comprises SR-TENG, power management circuit (PMC), wireless sensors, wireless monitoring terminals, etc. Its detailed working principle is illustrated in Fig. S21. PMC is used to enhance the energy output efficiency (Fig. 5B). Figure 5C demonstrates the effect of using PMC to accelerate the charging of the capacitor. With PMC, a 100-μF capacitor is charged to 3.89 V in just 30 s. Compared to not using PMC, the charging efficiency is markedly improved, with the rate of stored energy increase being approximately 242 times faster. Figure S22 gives the performance of using the PMC to charge other large capacitors.

Figure 5D illustrates the picture of the wireless self-powered water temperature sensing node, utilizing SR-TENG. The detailed working processes are illustrated in Fig. 5E. A 2.2-mF capacitor charges to 5.1 V in 14.5 min, after which LTC 3588-1 automatically activates, and the commercial wireless water temperature sensor (DS18B20) begins to operate continuously and stably. The water temperature sensor collects data every 50 s and transmits the signal to the receiving terminal for real-time display (Movie S5). Figure 5F and Movie S6 demonstrate the practical application of the wireless water level early warning system. The 8-mF capacitor charges to 5.1 V within 33 min, after which LTC 3588-1 automatically activates, and the wireless water level sensor is successfully powered on, with the voltage dropping to 3.8 V. When the water level exceeds the threshold, the wireless water level sensor sends a signal. Upon receiving the signal, the early warning terminal triggers an alarm. The voltage curve and process of the wireless self-powered water quality sensing system are shown in Fig. 5G and Movie S7. The 8-mF capacitor charges to 6.5 V in 46 min to power the wireless water quality sensor, and the data are successfully transmitted to and displayed on the mobile phone via Bluetooth. The above research fully demonstrates the feasibility of SR-TENG to energy harvesting and self-powered sensing in the water environment.

## Conclusion

In this paper, an interface SR-TENG is proposed. By concurrently controlling the interface contact state and contact force, the triboelectric materials achieve adaptive dynamic interfacial contact and micro-sliding under minimal normal pressure, enabling TENG to achieve ultra-high electrical output performance and outstanding durability. Experimental results show that, in comparison with conventional TENGs, the output energy of SR-TENG is enhanced by 13 times, and the frictional force is reduced by up to 72.5 times. Based on this, a galloping-driven integrated prototype based on SR-TENG has been developed, which is highly appropriate for the collection of water flow energy. The peak power density of this device attains 242.4 mW/m^2^, and after continuous operation in the water flow for 10 h, the output current still retains 97.6%. Furthermore, a PMC was developed to boost SR-TENG’s electrical output, enhancing its energy storage speed by 242 times. Finally, a wireless self-powered sensing system was constructed to support the monitoring of the water environment (including water temperature, water level, and water quality parameters), verifying the potential of SR-TENG for use as a self-powered sensing node in environmental monitoring. This research offers a novel approach to TENG’s practical application in renewable energy utilization and environmental protection.

## Materials and Methods

### Fabrication of SR-TENG

Figure 1C displays the structure of SR-TENG. The driver part, follower part, limiter, and housing are all fabricated using 3D printing (model: Raise3D Pro3 Plus HS; layer height: 0.05 mm; print speed: 15 to 300 mm/s), with 1.75-mm-diameter polylactic acid (PLA) as the material. The guide rails are fixed to the inner wall of the housing. Slider 1 (MGN7C) and self-regulating slider (SGB10) on the guide rails are connected to the driver part and follower part, respectively. The carbon fiber substrate of the freestanding layer (length: 80 mm; width: 80 mm; thickness: 1.5 mm) is connected to the chute of the driver part via an interference fit. Sponges (length: 80 mm; width: 50 mm; thickness: 0.5 mm) are attached to both sides of each substrate. Then, 30-μm-thick FEP films are attached to the surfaces of the 0.5-mm-thick sponges. The carbon fiber substrate (length: 80 mm; width: 80 mm; thickness: 2 mm) is connected to the chute of the follower part via an interference fit. Sponges (length: 80 mm; width: 50 mm; thickness: 1 mm) are attached to 2 faces of substrate. The outermost baseplate requires the application of sponge on only one side. Next, a layer of copper foil electrode with a thickness of 60 μm is attached to the sponge top. Finally, the 25-μm-thick Nylon films are placed on the electrodes.

### Fabrication of GDSR-TENG

Figure 4A illustrates the construction of GDSR-TENG prototype. The primary components consist of an SR-TENG, 2 compression springs, a frame, and a semi-circular bluff body. The frame and the semi-circular bluff body (diameter: 80 mm; length: 320 mm) are manufactured using 3D printing technology, with premium PLA as the material. The bluff body mounts rigidly at the frame end. The frame is used to ensure consistent vertical motion. The compression springs (wire diameter: 1 mm; outer diameter: 30 mm; length: 80 mm), made of stainless steel, are symmetrically positioned to securely connect the bottom driving component of SR-TENG to the frame. These springs transduce the bluff body’s motion into elastic potential energy, subsequently driving SR-TENG to generate electricity.

### Electrical measurements of the device

SR-TENG is driven by a linear motor (B01-37 × 166/160, LinMot). The driving force and interface contact force by the device are measured using a tension pressure sensor (ZZ010-050A, Zhizhan Measurement & Control). The frictional drag of the self-regulating slider is characterized using a push–pull force gauge (SMF-30, SanLiang). An electrometer (Keithley 6514) measures the output charge and current. An oscilloscope (MDO34, Tektronix) is used to obtain the output voltage. Various water flow velocities are generated by 6 water pumps (AQ12000DP, Huitian), and the specific velocities are measured using a flow velocity meter (LS300-A, Zhuoma). The amplitude and frequency of the blunted body are determined using a high-speed camera (AX100, Nikon). The surface morphology images of Nylon film and FEP film are obtained using a scanning electron microscope (G300, ZEISS).

## Data Availability

The data that support the findings of this study are available from the corresponding authors upon reasonable request.

## Supplementary Materials

Supplementary 1 Figs. S1 to S22 Tables S1 to S3 Notes S1 and S2 Movies S1 to S7
