# Supplementary material for: Boosting Output Performance of Triboelectric Nanogenerator via Interface Self-Regulation Strategy
Source: Research (Wash D C). 2025 Sep 25;8:0906. doi: 10.34133/research.0906 (PMC12463547; doi:10.34133/research.0906)
Supplement: Supplementary 1 — Figs. S1 to S22 Tables S1 to S3 Notes S1 and S2 Movies S1 to S7 [file research.0906.f1.zip › 4-Supplementary Materials.docx]

Supplementary Materials for

**Boosting Output Performance of Triboelectric Nanogenerator via Interface Self-Regulation Strategy**

Yanrui Zhao^1,2^†, Yuming Feng^1,2^†, Qi Gao^3^†, Hengyu Li^1,2^, Xin Guo^1,2^, Jianlong Wang^1,2^, Xinxian Wang^1,2^, Lu Dong^1^, Yang Yu^1,2*^, Zhong Lin Wang^1,3*^, Tinghai Cheng^1,2,3*^

^1^Beijing Institute of Nanoenergy and Nanosystems, Chinese Academy of Sciences, Beijing 101400, China

^2^School of Nanoscience and Engineering, University of Chinese Academy of Sciences, Beijing 100049, China

^3^Guangzhou Institute of Blue Energy, Knowledge City, Huangpu District, Guangzhou 510555, China

†These authors contributed equally to this work.

^*^Address correspondence to: yuyang@binn.cas.cn; zhong.wang@mse.gatech.edu; chengtinghai@binn.cas.cn.

**Contents**

**Supplementary Figures:**

**Fig. S1.** Working principle of SR-TENG.

**Fig. S2.** Force analysis of SR-TENG during operation.

**Fig. S3.** The different molecular distances of the FEP/Nylon interface model.

**Fig. S4.** The experimental system of SR-TENG and CF-TENG.

**Fig. S5.** Two-dimensional diagram of size parameters of SR-TENG.

**Fig. S6.** Output performance of SR-TENG under varying contact forces.

**Fig. S7.** Output performance of CF-TENG under varying contact forces.

**Fig. S8.** Comparison of output power between CF-TENG and SR-TENG.

**Fig. S9.** Output current curves of CF-TENG and SR-TENG under different resistances.

**Fig. S10.** Comparison of output performance of different triboelectric material pairs.

**Fig. S11.** Output charge of SR-TENG and CF-TENG during consecutive operations.

**Fig. S12.** Driving force of SR-TENG under different slider drags.

**Fig. S13.** Output current of SR-TENG with different (A) freestanding layer substrate thicknesses, (B) distances between two electrodes, and (C) layout angles.

**Fig. S14.** Output current of SR-TENG with different (A) electrode areas and (B) sliding distances.

**Fig. S15.** Output voltage of SR-TENG at different excitation frequencies.

**Fig. S16.** Photograph of the designed prototype.

**Fig. S17.** Movement process of the designed prototype under the action of water flow.

**Fig. S18.** Output voltage of the designed prototype at various flow speeds.

**Fig. S19.** Performance comparison between this work and reported works.

**Fig. S20.** Output currents of the designed prototype under different load resistances.

**Fig. S21.** Working principle of water environmental wireless self-powered sensing system.

**Fig. S22.** Capacitor charging curve with PMC.

**Supplementary Tables:**

**Table S1.** Comparison of peak power density of SR-TENG with other reported works.

**Table S2.** Theoretical values of *F*_n_ and *F* (including *F*_P_ and *F*_t_) at electrode arrangement angle *θ* of 60°corresponding to different *F*_f_ values.

**Table S3.** Theoretical values of *F*_n_ and *F* (including *F*_P_ and *F*_t_) at *F*_f_ of 1.2 N corresponding to different electrode arrangement angle *θ*.

**Supplementary Notes:**

**Note S1.** Equations of force analysis of SR-TENG.

**Note S2.** Measurement system of SR-TENG and CF-TENG.

**Supplementary Movies:**

**Movie S1.** The demonstration animation of interface self-regulation concept.

**Movie S2.** The effect of SR-TENG on increasing output performance.

**Movie S3.** The effect of SR-TENG on reducing the driving force.

**Movie S4.** Working process of GDSR-TENG prototype.

**Movie S5.** Self-powered wireless water temperature sensing based on the SR-TENG.

**Movie S6.** Self-powered wireless water level monitoring based on the SR-TENG.

**Movie S7.** Self-powered wireless water quality sensing based on the SR-TENG.


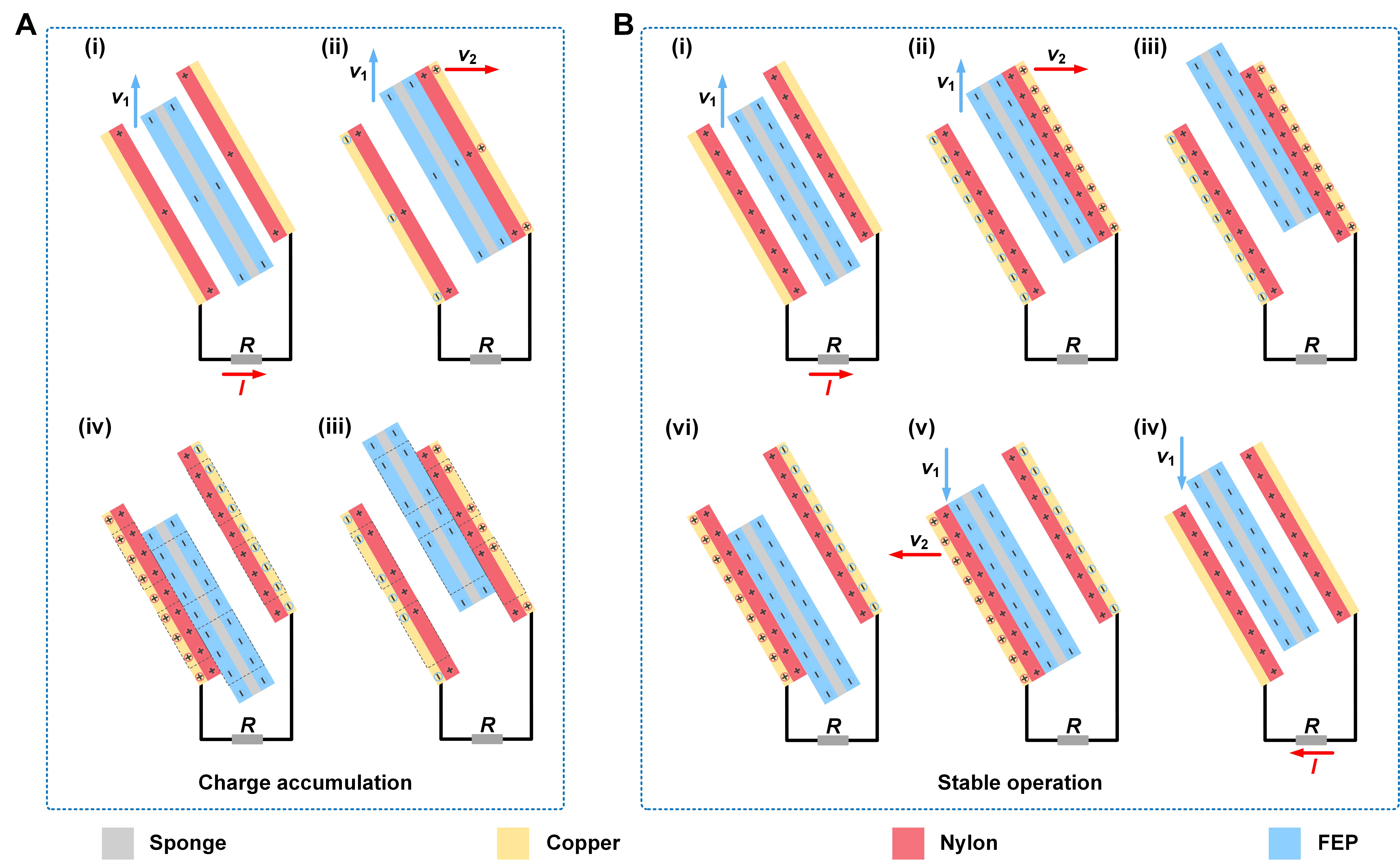


**Fig. S1.** Working principle of SR-TENG. (A) Charge accumulation stage. (B) Stable operation stage.

**
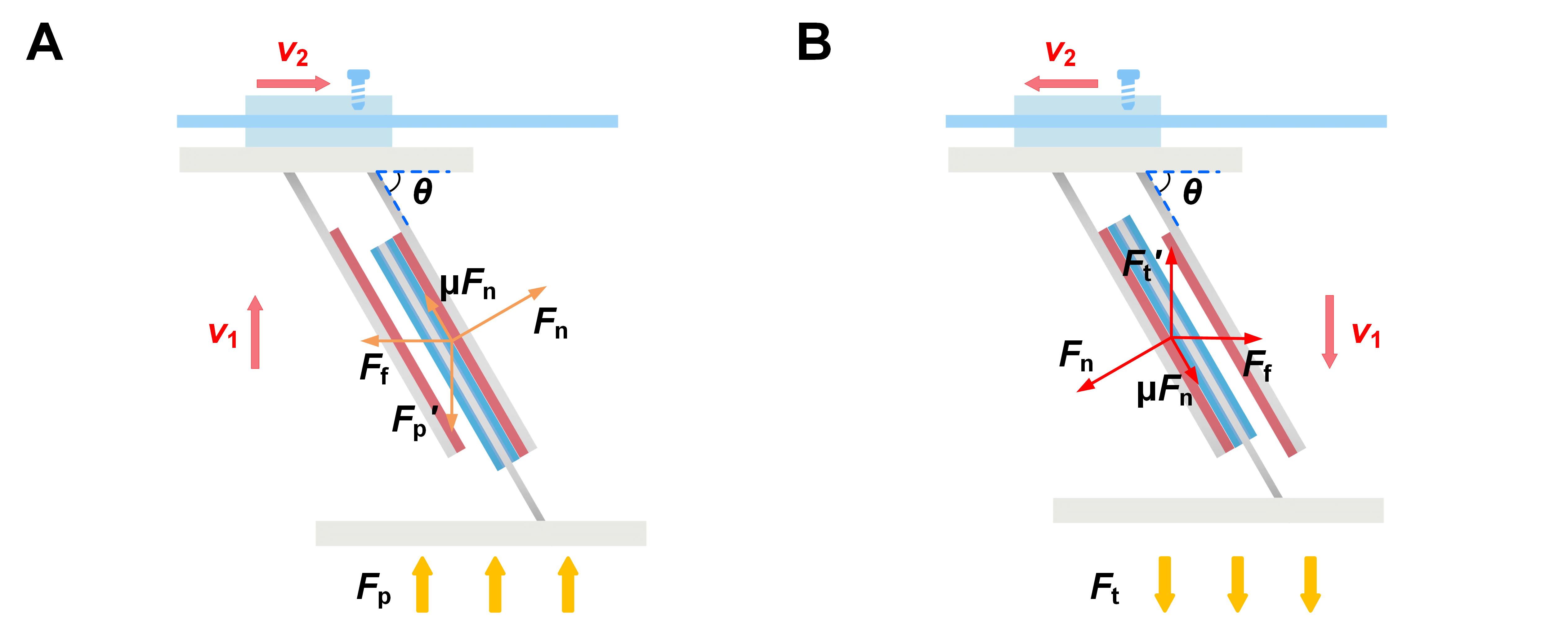
**

**Fig. S2.** Force analysis of SR-TENG during operation. (A) During the upward movement. (B) During the downward movement.


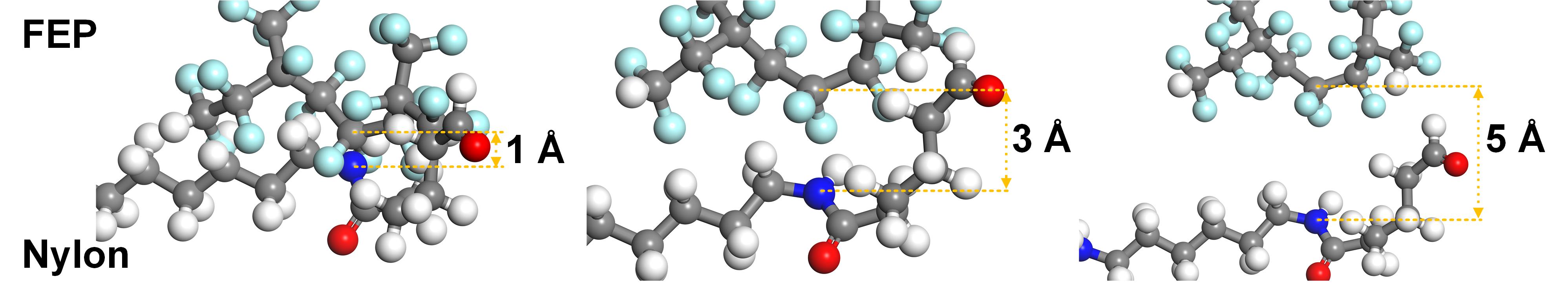


**Fig. S3.** The different molecular distances of the FEP/Nylon interface model.


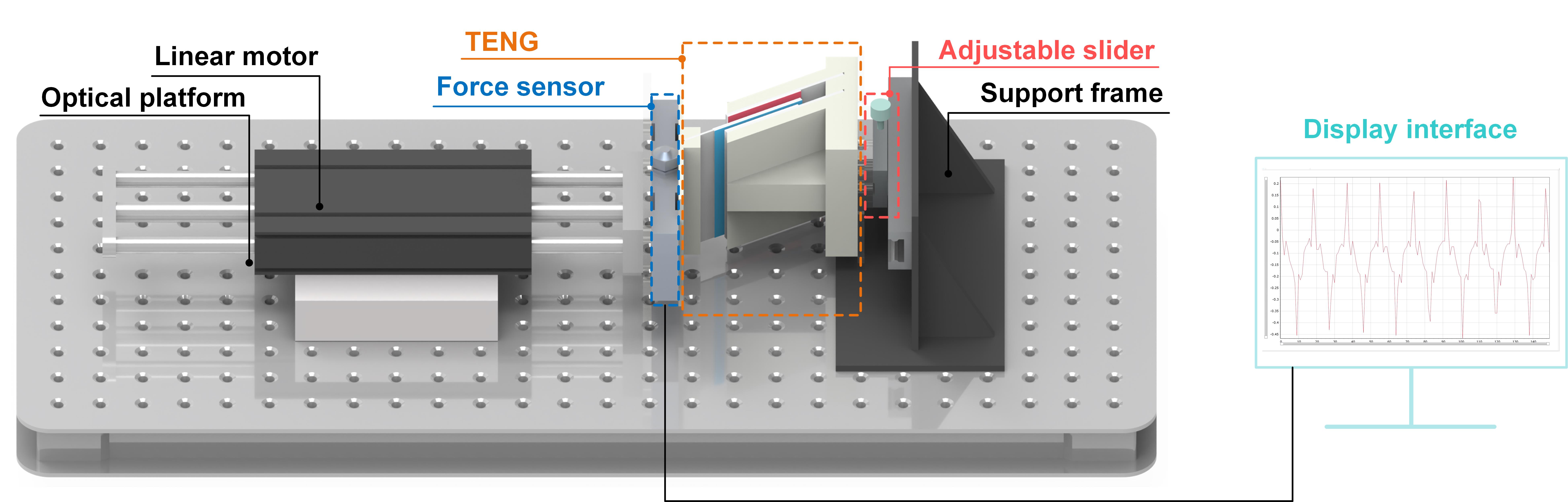


**Fig. S4.** The experimental system of SR-TENG and CF-TENG.


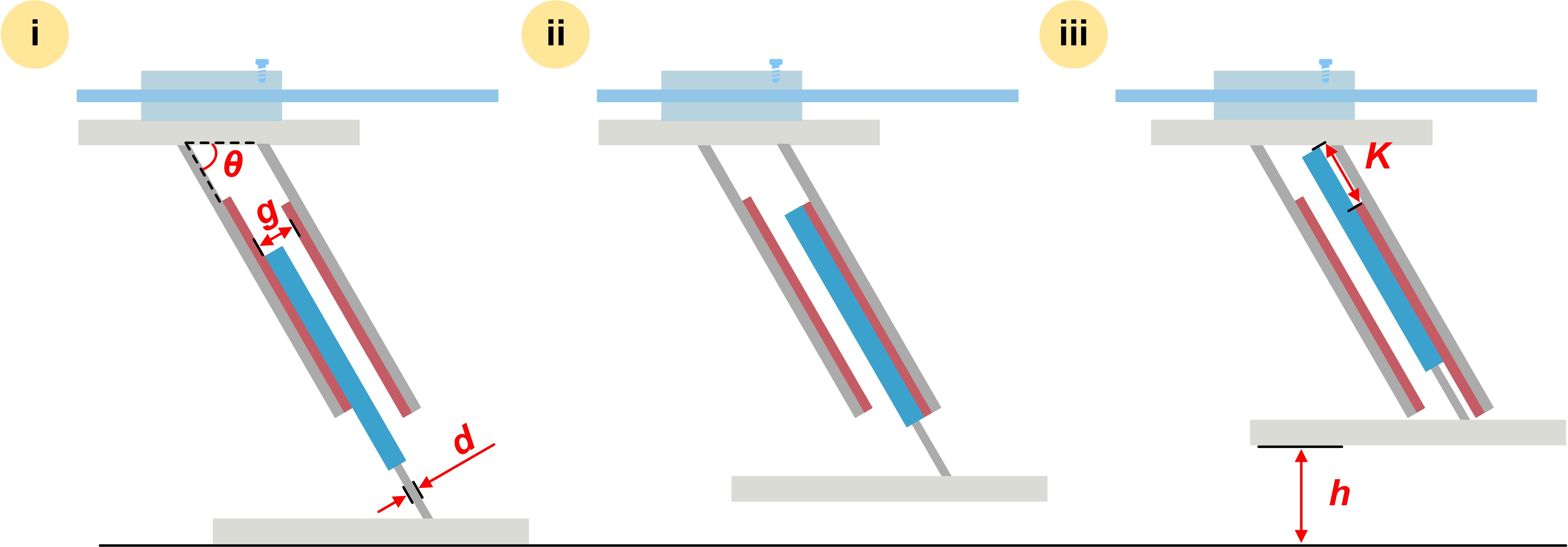


**Fig. S5.** Two-dimensional diagram of size parameters of SR-TENG.

**
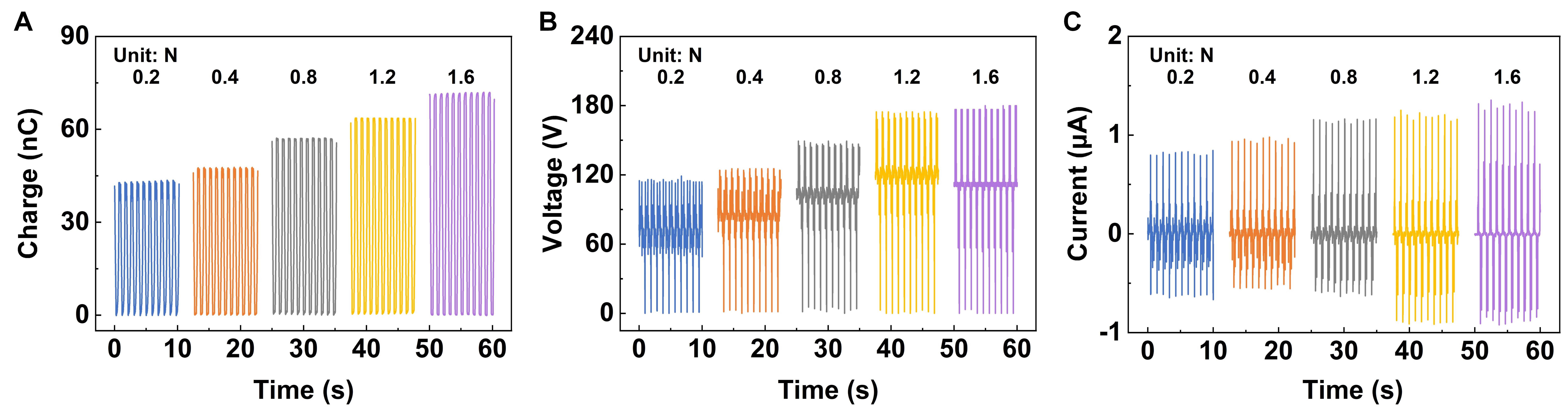
**

**Fig. S6.** Output performance of SR-TENG under varying contact forces. (A) Output charge. (B) Output voltage. (C) Output current.


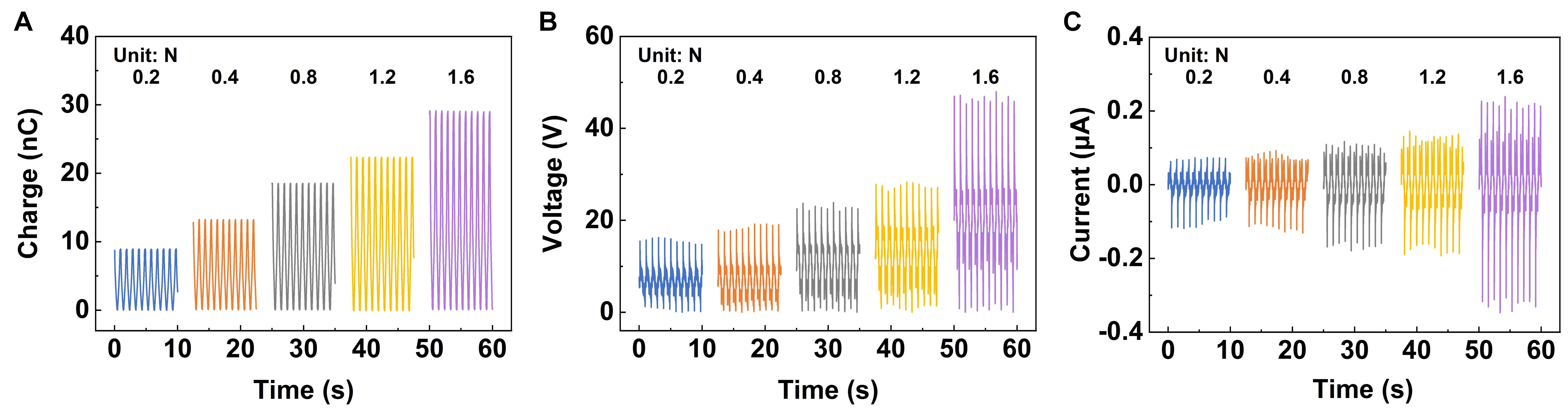


**Fig. S7.** Output performance of CF-TENG under varying contact forces. (A) Output charge. (B) Output voltage. (C) Output current.


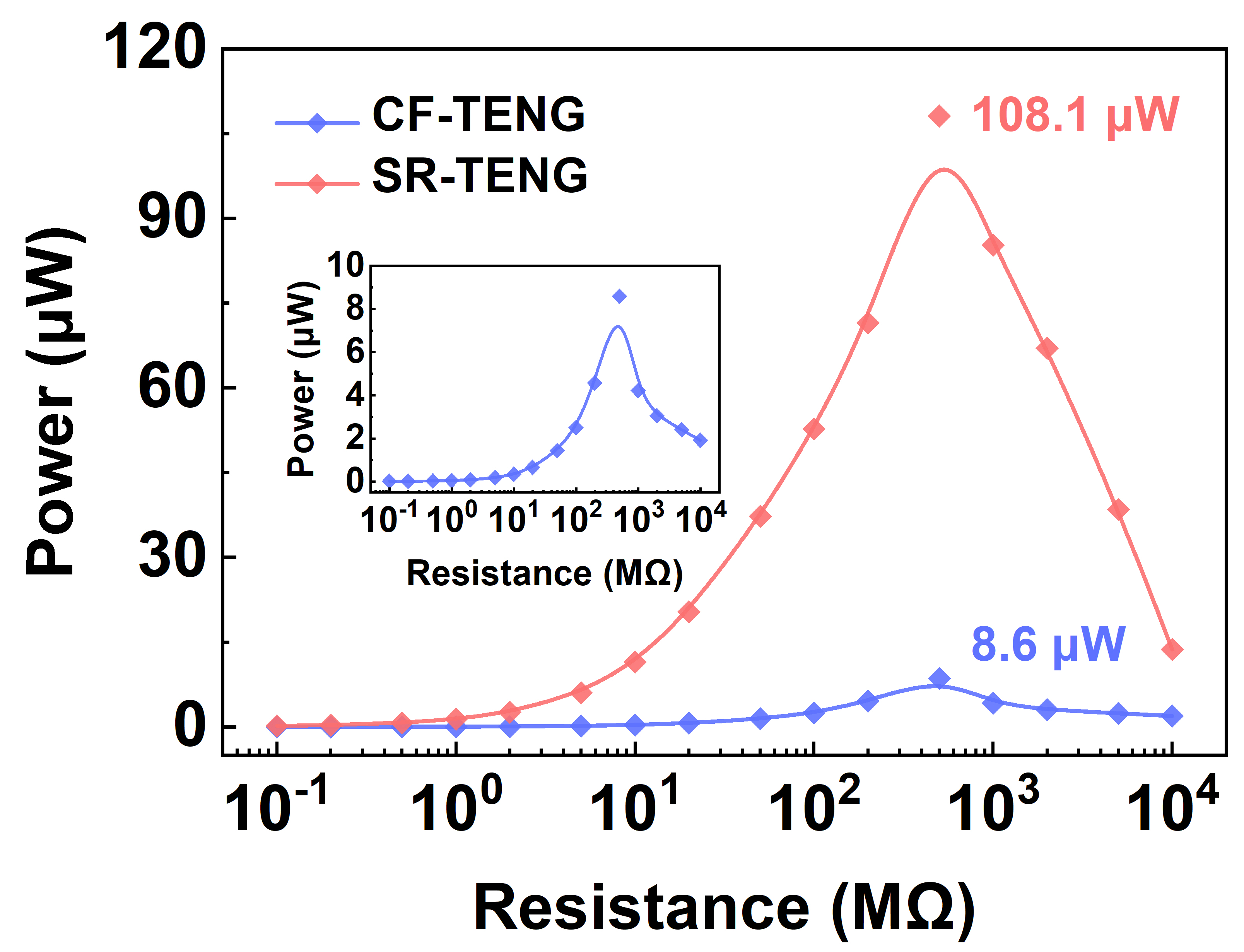


**Fig. S8.** Comparison of output power between CF-TENG and SR-TENG.


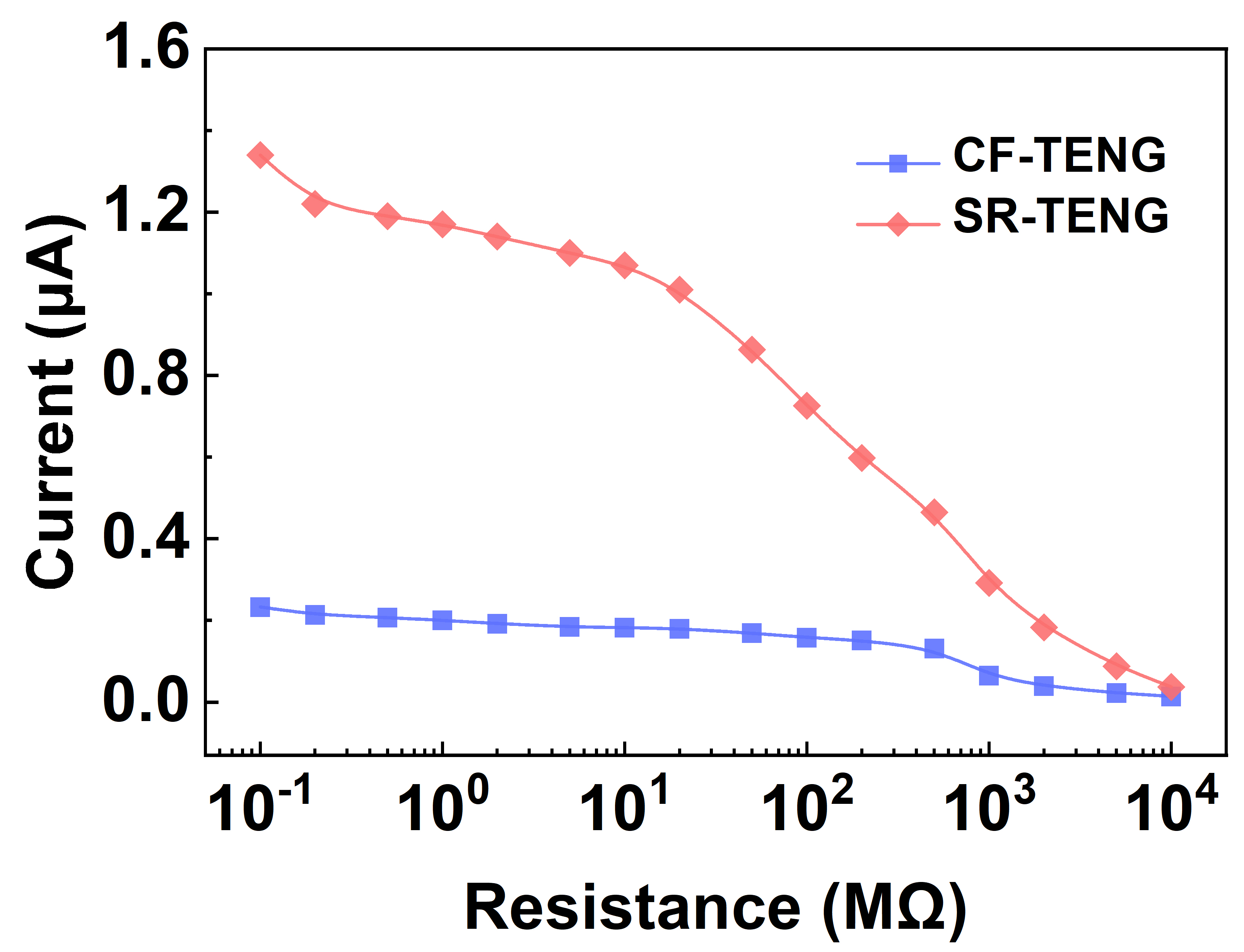


**Fig. S9.** Output current curves of CF-TENG and SR-TENG under different resistances.


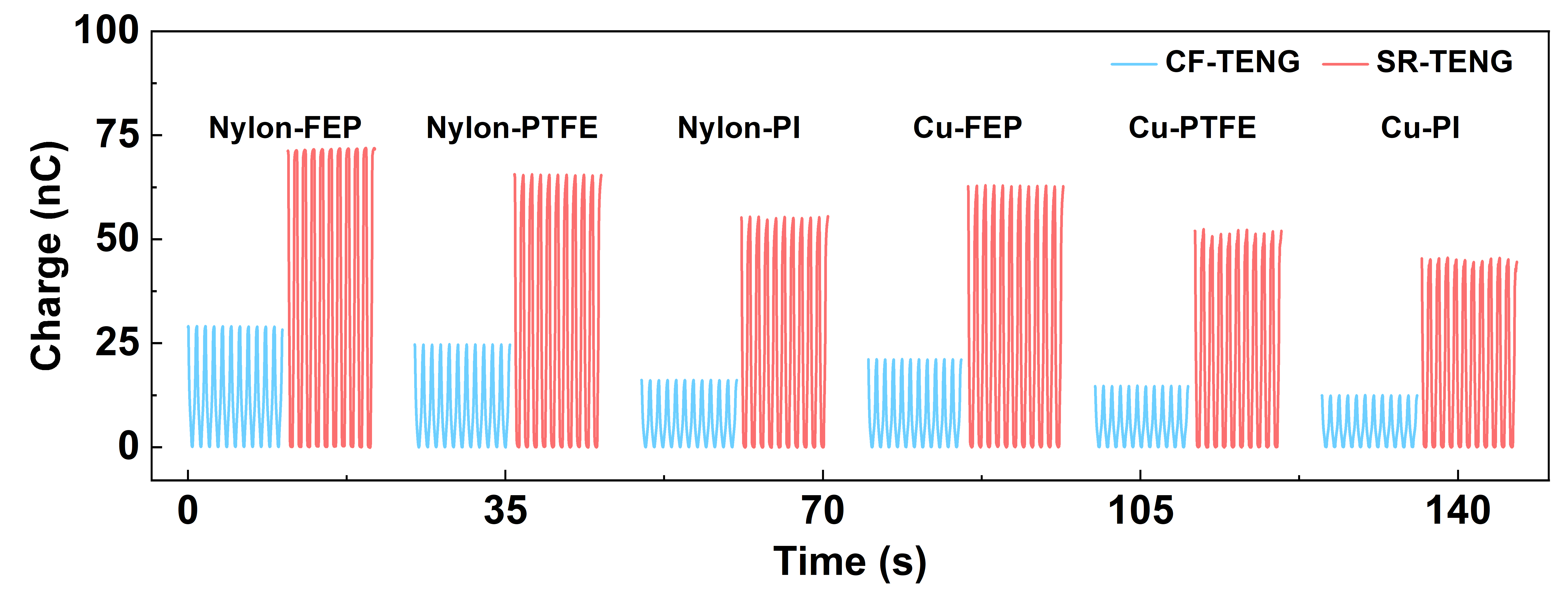


**Fig. S10.** Comparison of output performance of different triboelectric material pairs.


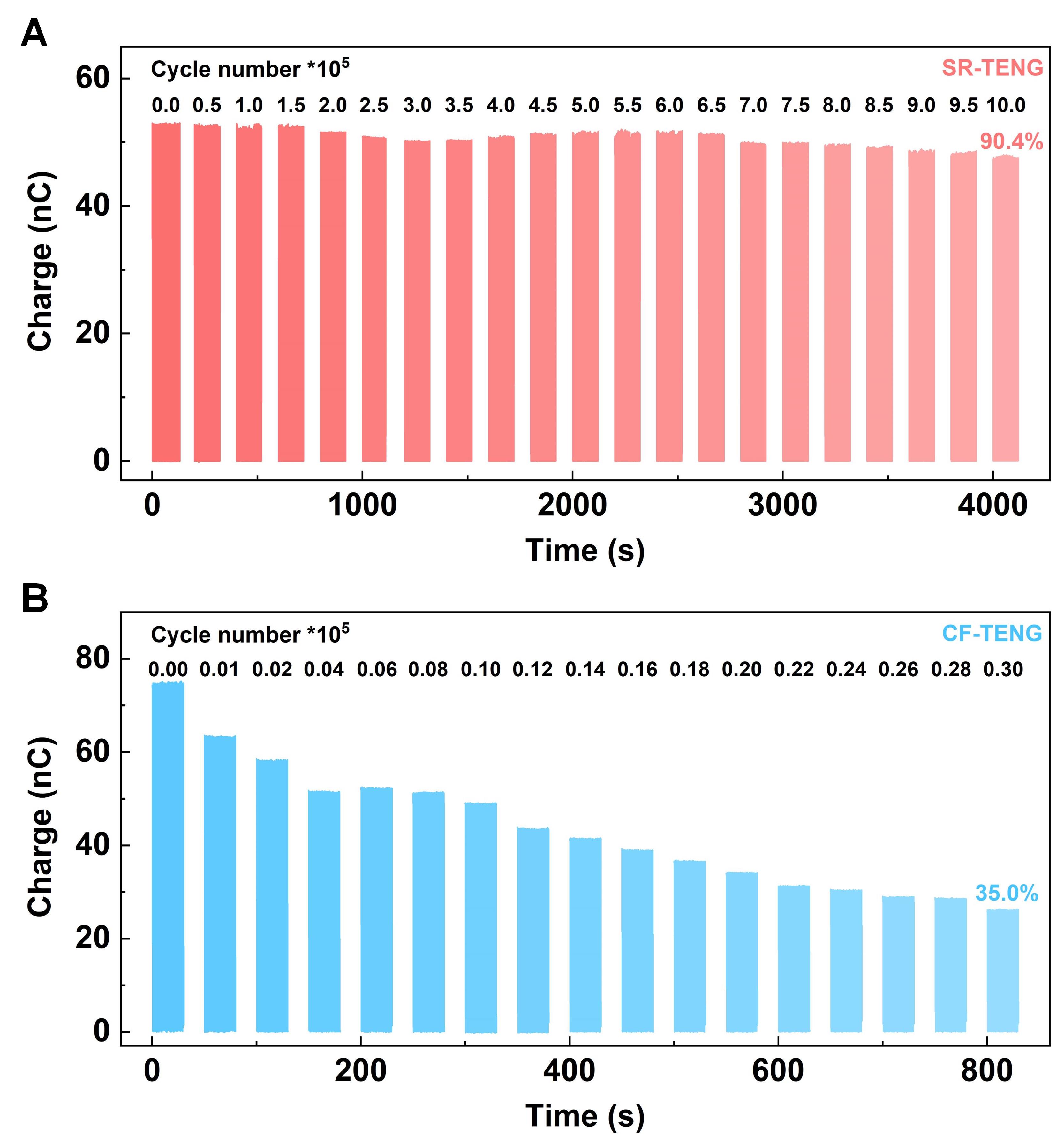


**Fig. S11.** Output charge of SR-TENG and CF-TENG during consecutive operations.


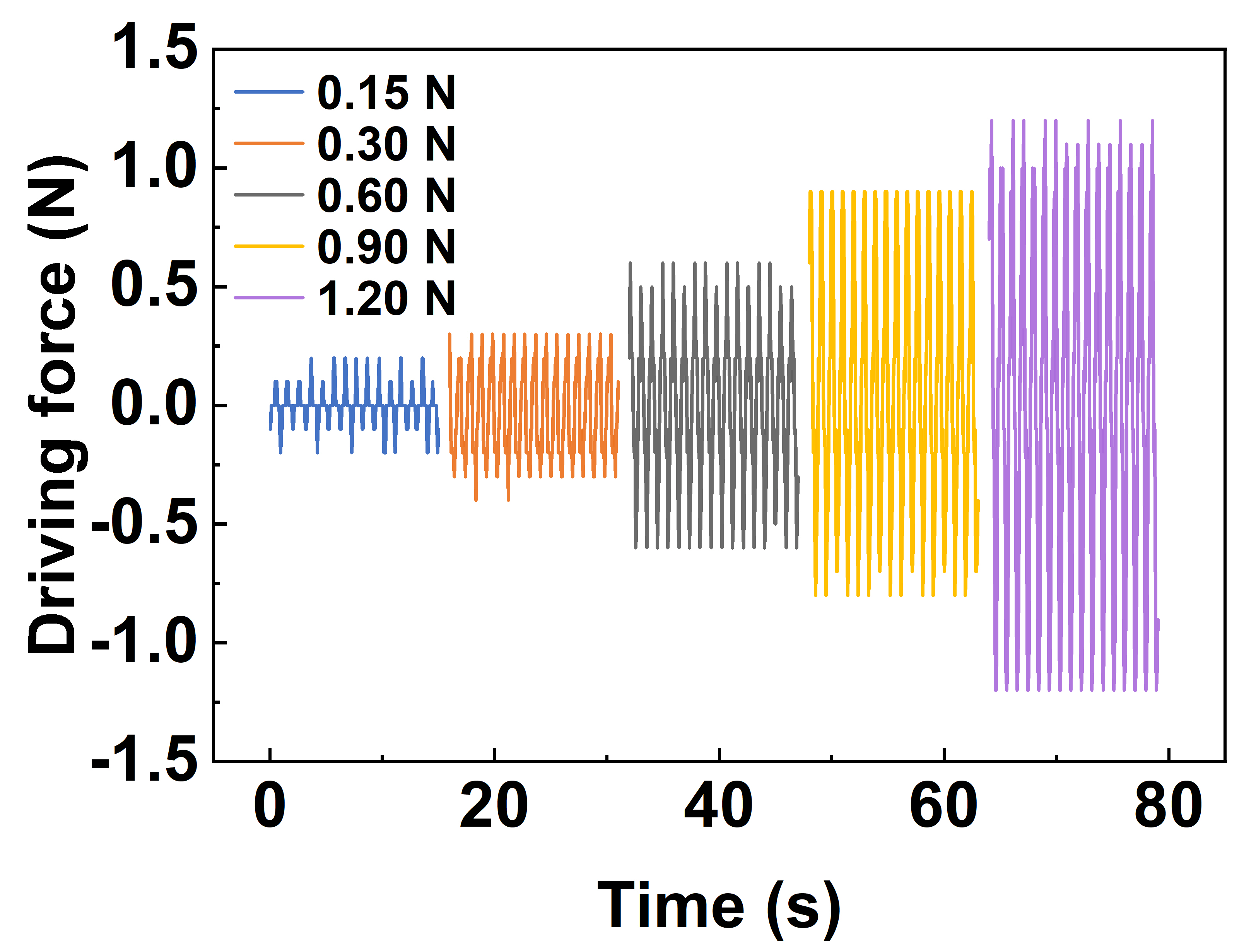


**Fig. S12.** Driving force of SR-TENG under different slider drags.


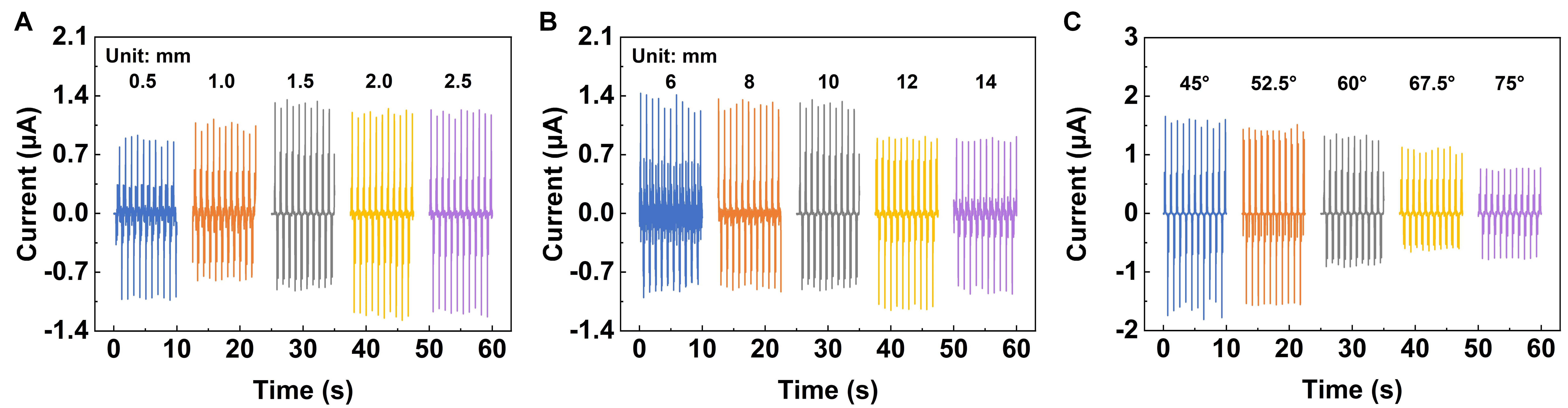


**Fig. S13.** Output current of SR-TENG with different (A) freestanding layer substrate thicknesses, (B) distances between two electrodes, and (C) layout angles.


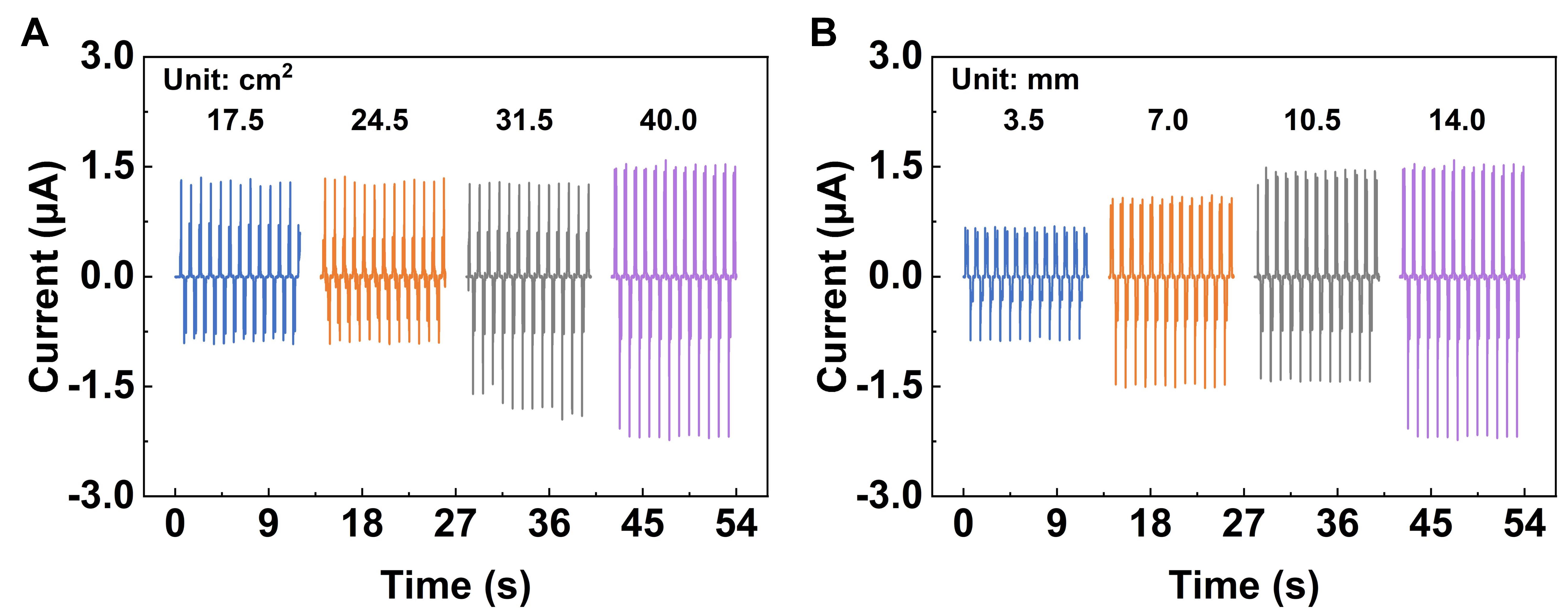


**Fig. S14.** Output current of SR-TENG with different (A) electrode areas and (B) sliding distances.


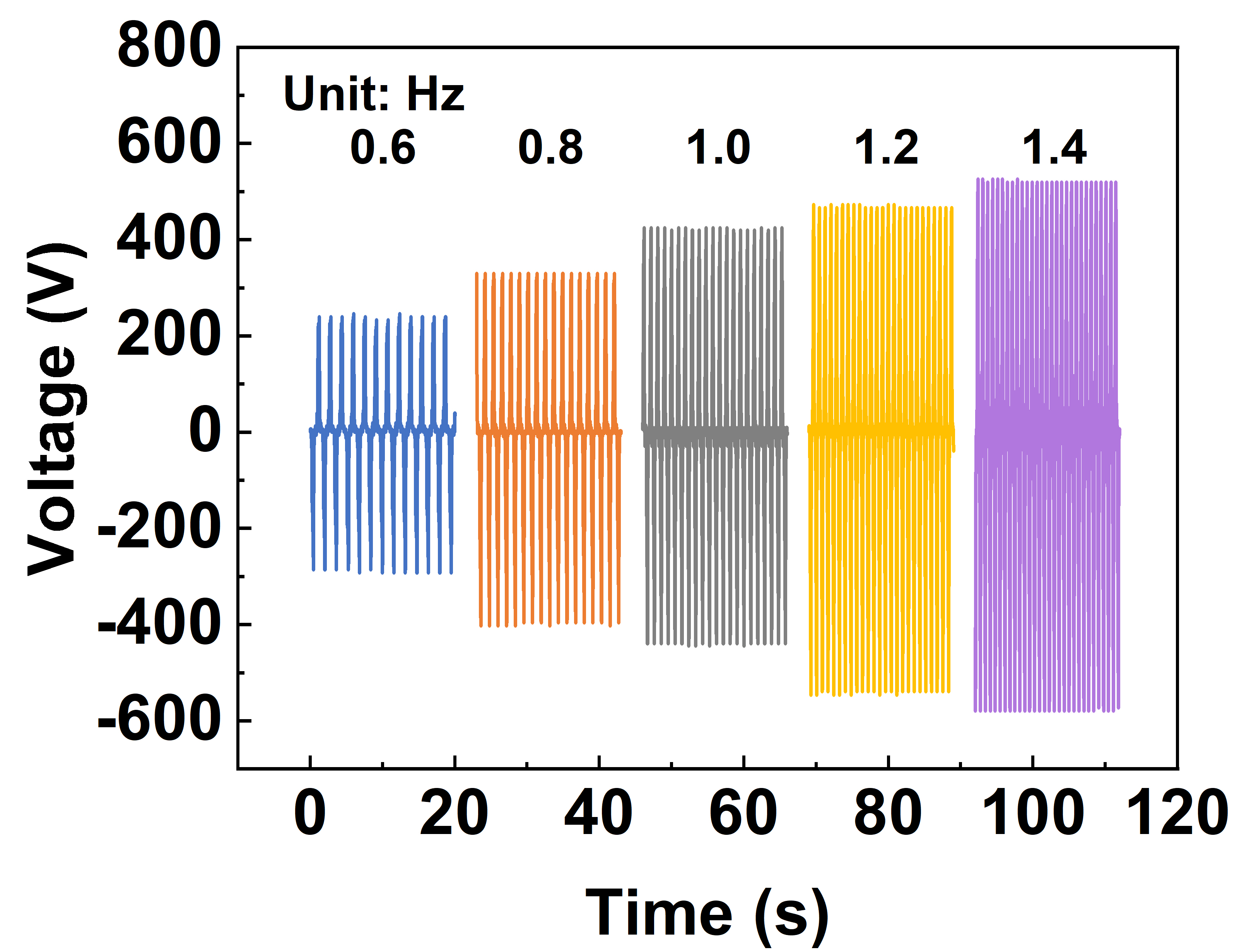


**Fig. S15.** Output voltage of SR-TENG at different excitation frequencies.


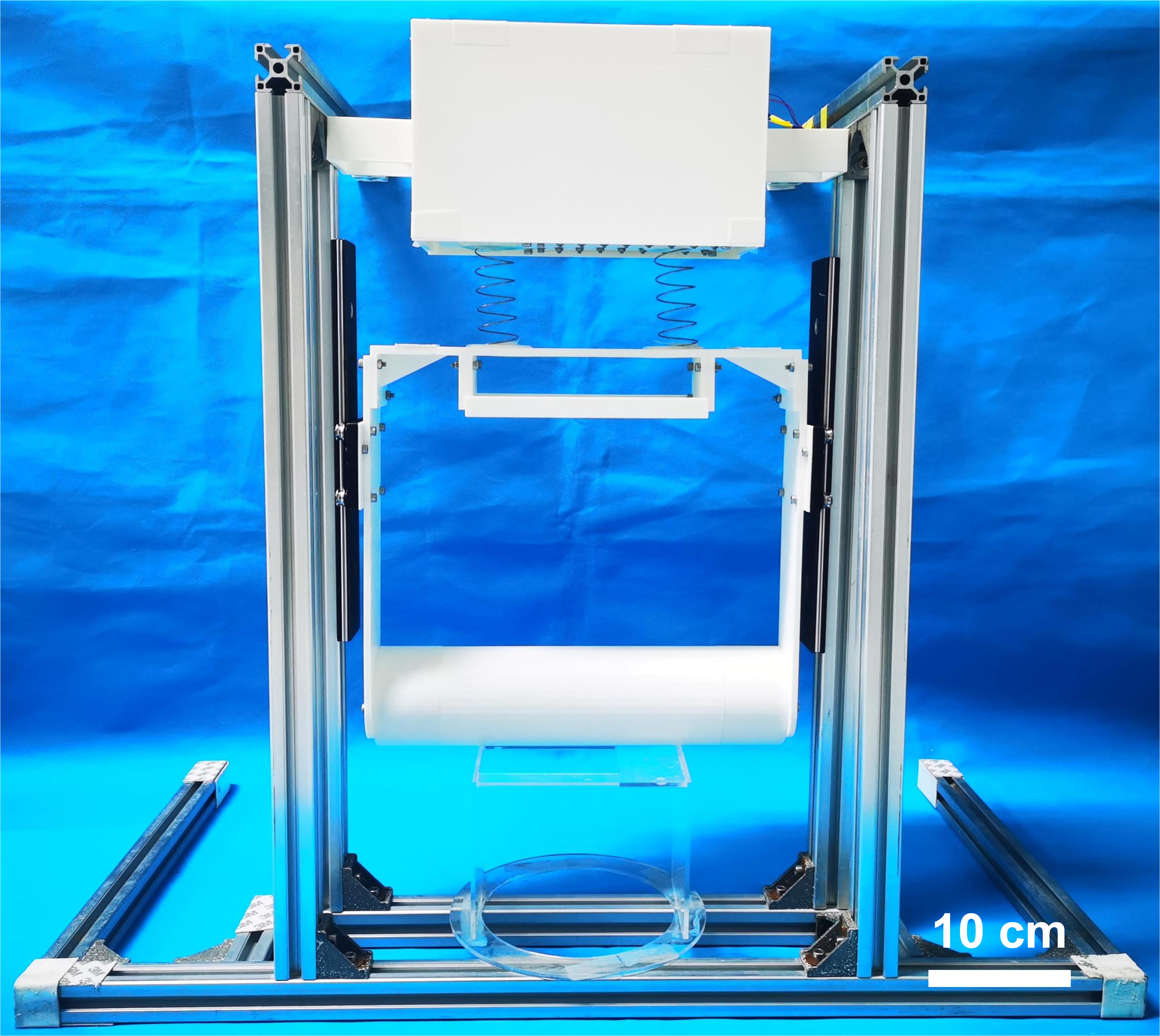


**Fig. S16.** Photograph of the designed prototype.


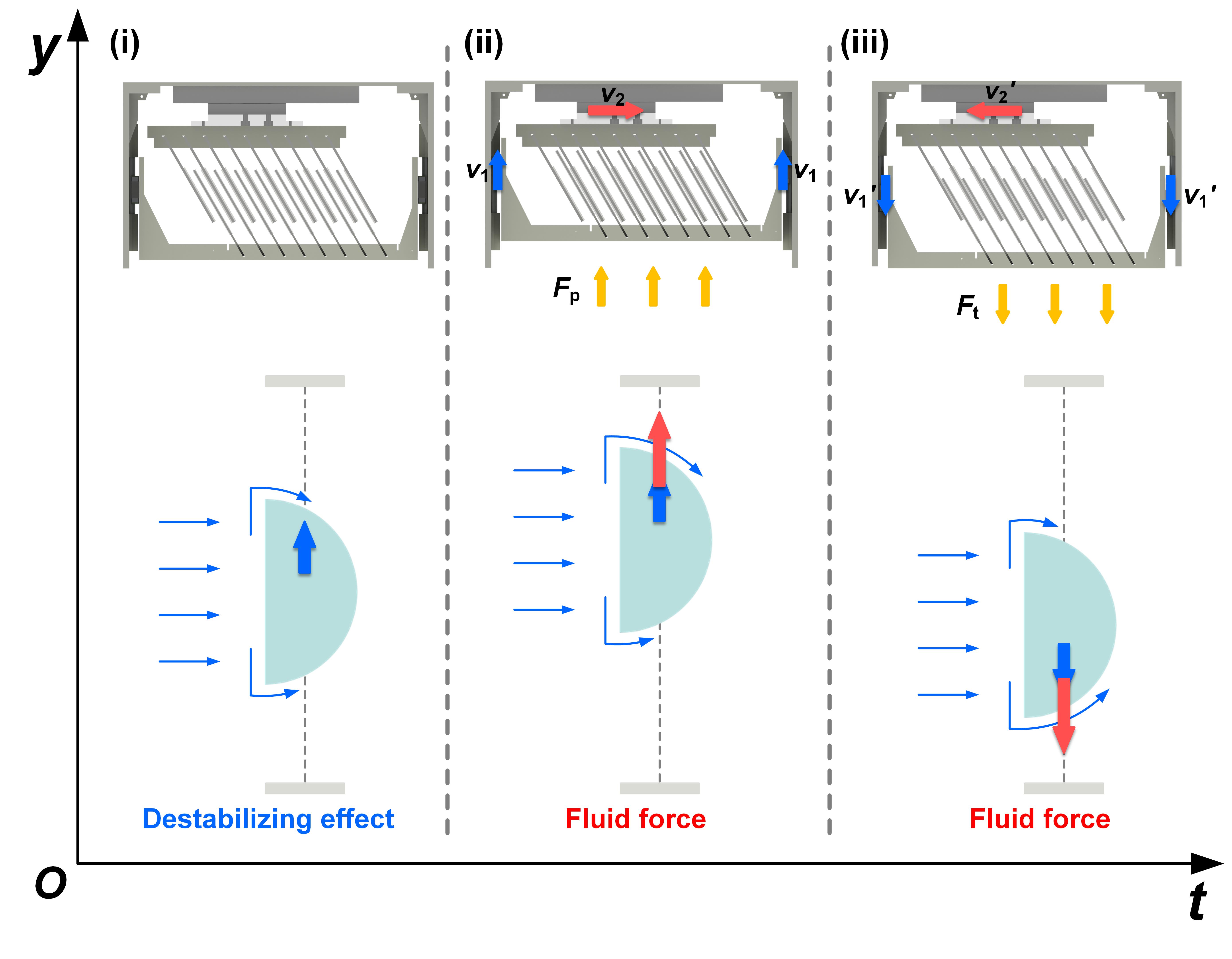


**Fig. S17.** Movement process of the designed prototype under the action of water flow.


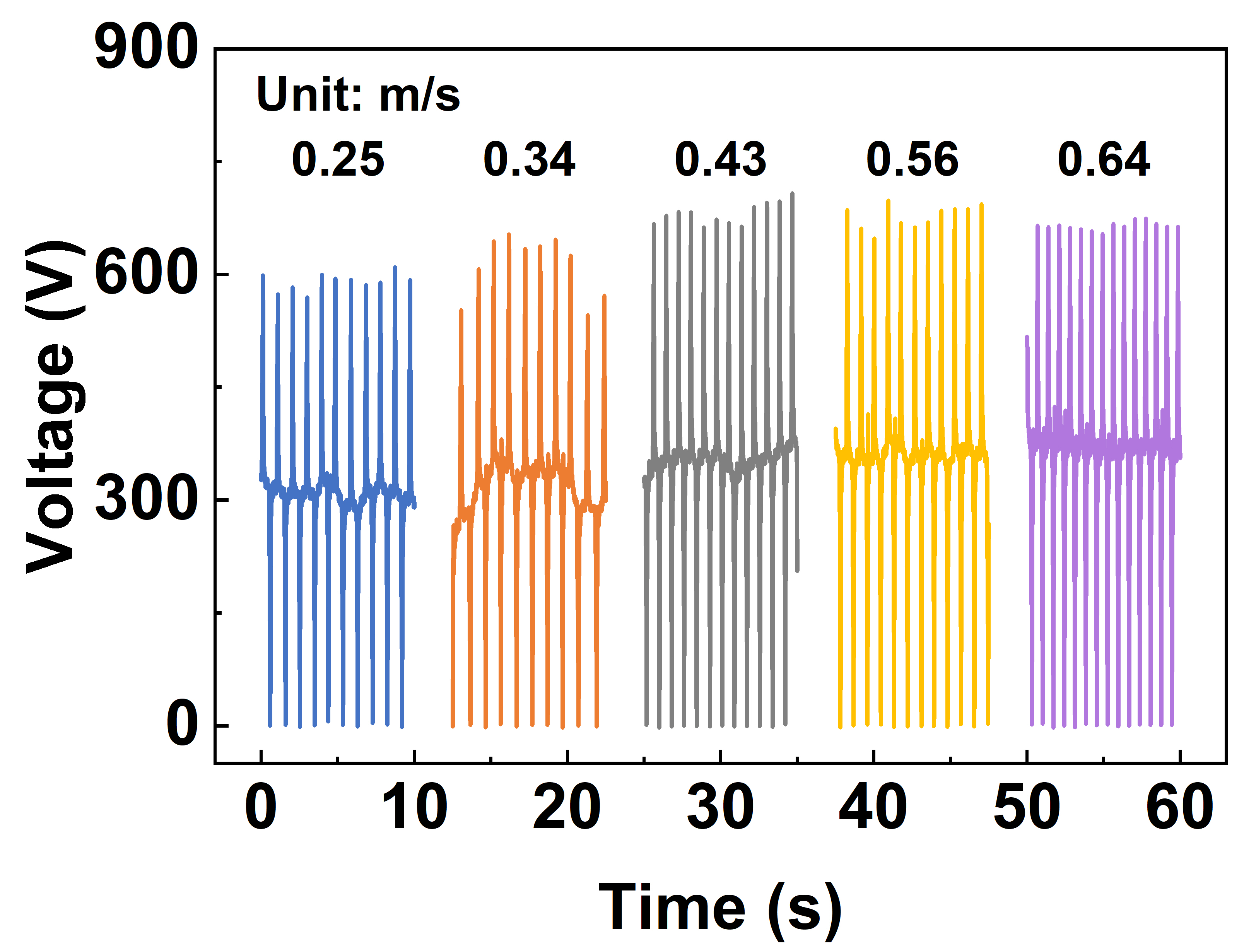


**Fig. S18.** Output voltage of the designed prototype at various flow speeds.


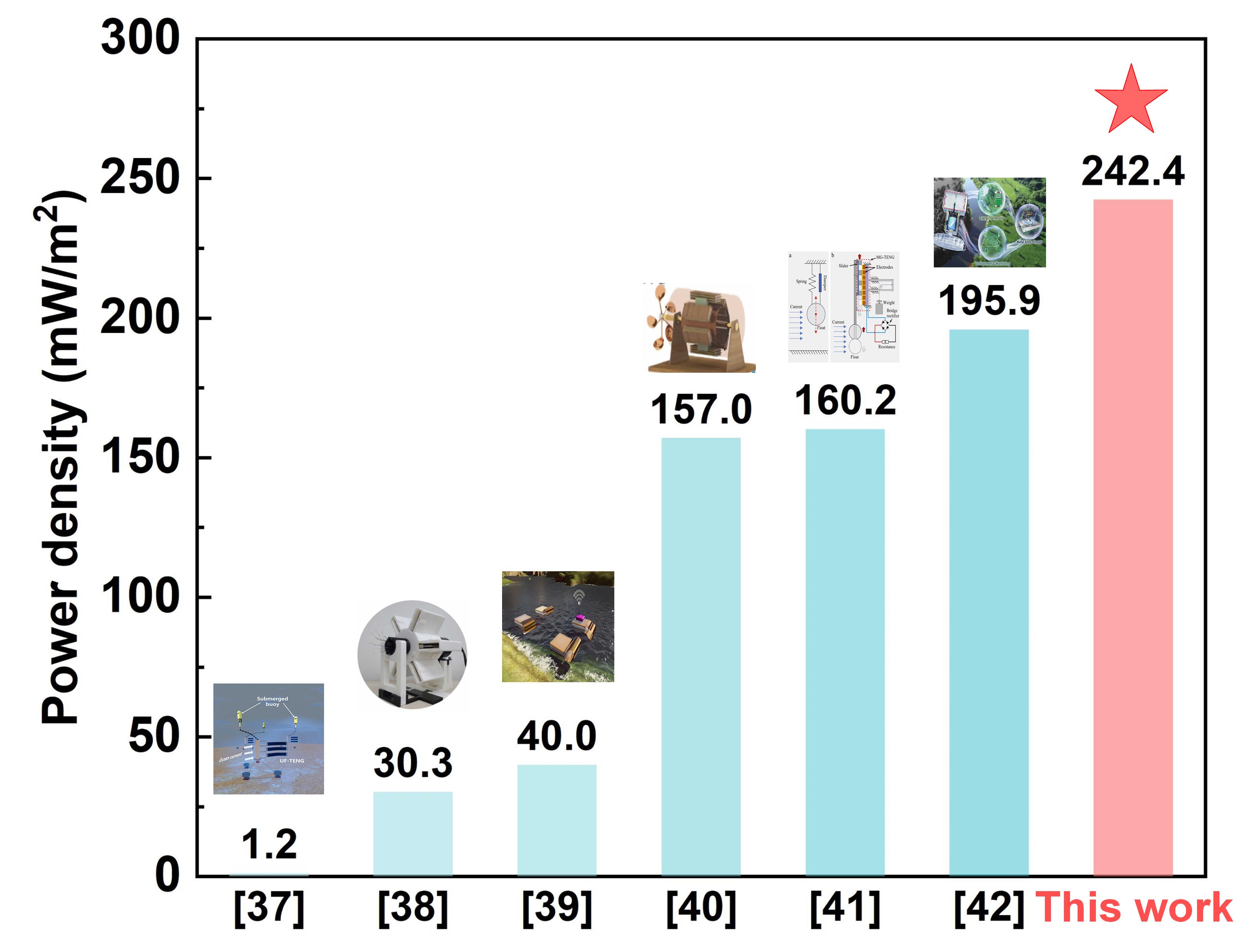


**Fig. S19.** Performance comparison between this work and reported works.


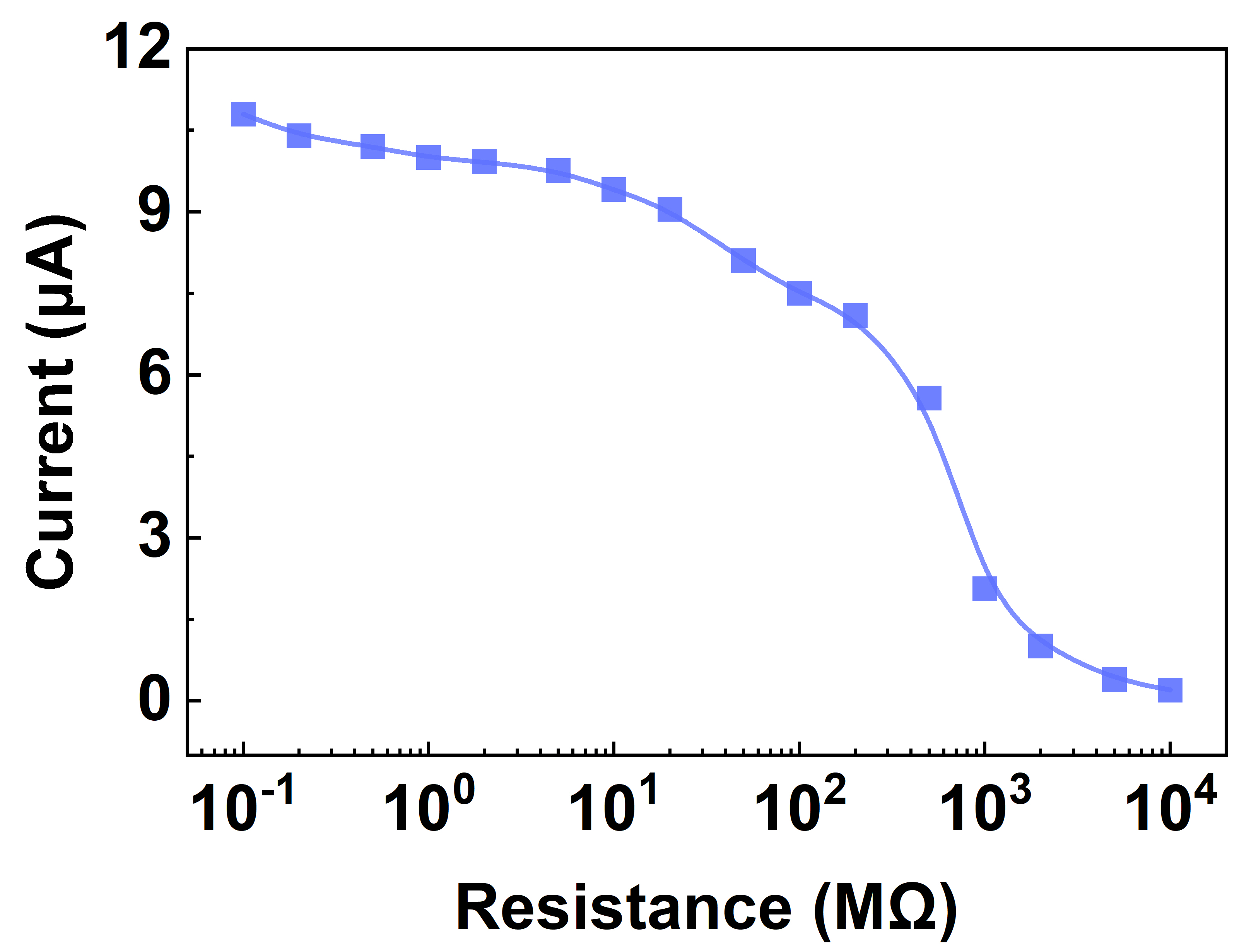


**Fig. S20.** Output currents of the designed prototype under different load resistances.


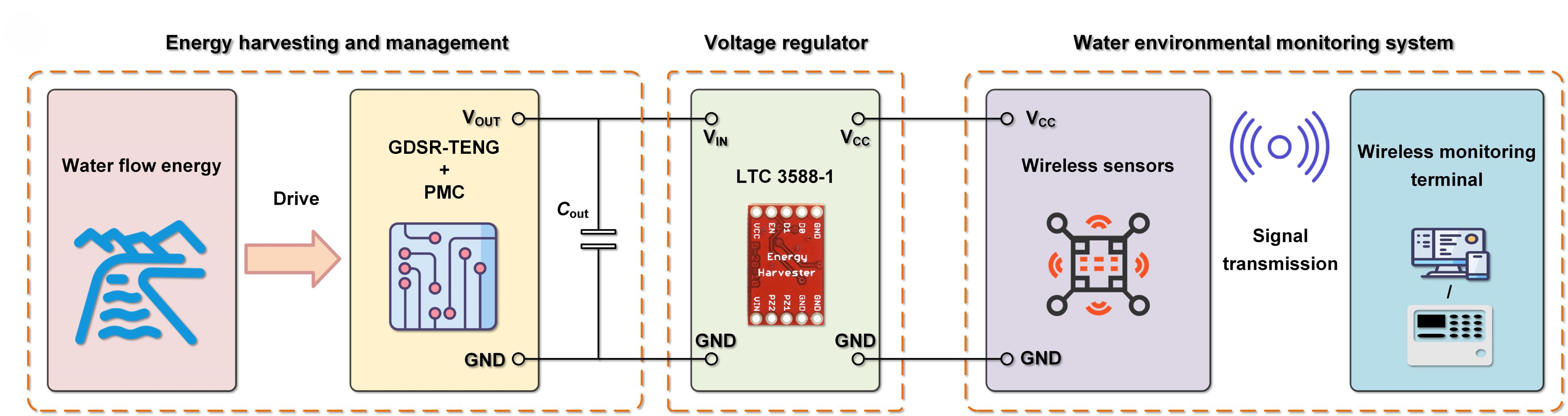


**Fig. S21.** Working principle of water environmental wireless self-powered sensing system.


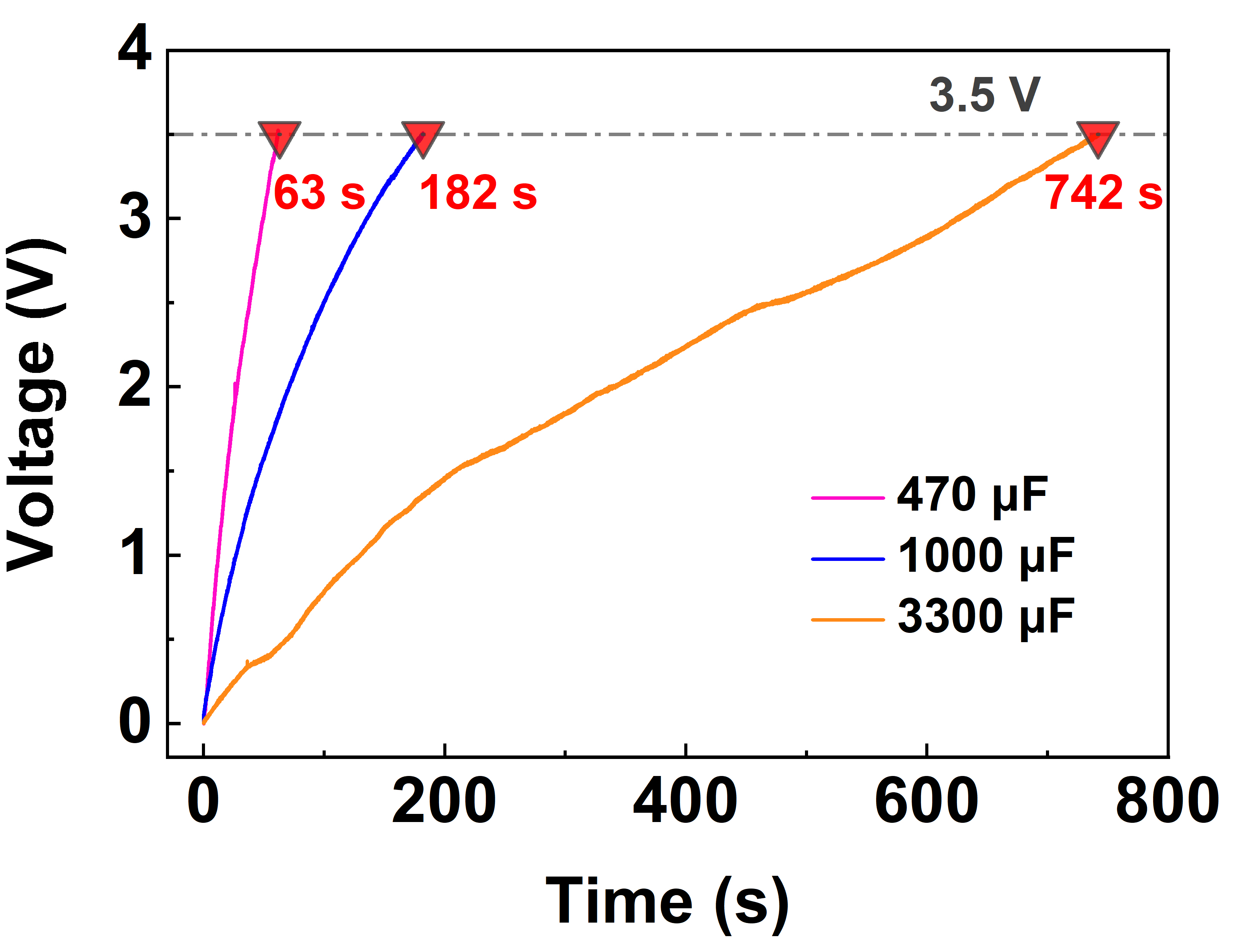


**Fig. S22.** Capacitor charging curve with PMC.

**Table S1.** Comparison of peak power density of SR-TENG with other reported works.

| Serial  number | Peak power density  (mW/m^2^) | Reference |
| --- | --- | --- |
| 1 | 1.2 | UF-TENG^1^ |
| 2 | 30.3 | RS-TENG^2^ |
| 3 | 40.0 | AS-TENG^3^ |
| 4 | 157.0 | RC-TENG^4^ |
| 5 | 160.2 | MG-TENG^5^ |
| 6 | 195.9 | MLS-TENG^6^ |
| 7 | 242.4 | This work |

**Table S2.** Theoretical values of *F*_n_ and *F* (including *F*_P_ and *F*_t_) at electrode arrangement angle *θ* of 60°corresponding to different *F*_f_ values.

| *F*_f_ (N) | 0.15 | 0.30 | 0.60 | 0.90 | 1.20 |
| --- | --- | --- | --- | --- | --- |
| *F*_n_ (N) | 0.20 | 0.40 | 0.80 | 1.20 | 1.60 |
| *F* (N) | 0.14 | 0.28 | 0.57 | 0.85 | 1.14 |

**Table S3.** Theoretical values of *F*_n_ and *F* (including *F*_P_ and *F*_t_) at *F*_f_ of 1.2 N corresponding to different electrode arrangement angle *θ*.

| *θ* (°) | 45° | 52.5° | 60° | 67.5° | 75° |
| --- | --- | --- | --- | --- | --- |
| *F*_n_ (N) | 2.23 | 1.85 | 1.60 | 1.44 | 1.33 |
| *F* (N) | 1.96 | 1.48 | 1.14 | 0.87 | 0.65 |

**Note S1.** Equations of force analysis of SR-TENG.

The force analysis of the SR-TENG (taking upward movement as an example) can be expressed as shown in the equation:

$F_{\text{n}}=F_{\text{P}}^{'}\cos\theta+F_{\text{f}}\sin\theta$ (S1)

Where *F*_n_ is the normal pressure applied to the interface, *F*_P_^'^ is the the external driving force, *F*_f_ is the frictional resistance of the slider, and *θ* is the angle at which the electrodes are arranged.

$\mu F_{\text{n}}=F_{\text{p}}^{'}\sin\theta-F_{\text{f}}\cos\theta$ (S2)

Where *μ* is the friction coefficient between Nylon film and FEP film, the value is confirmed to be 0.24^7^, according to existing research.

The primary objectives of force analysis are twofold: first, to enhance comprehension of the operational mechanisms of SR-TENG; second, to precisely characterize experimental conditions by leveraging the interrelationships among variables *F*_n_, *F*_P_^'^, *F*_f_, and *θ*. The derived results are displayed in Table S2 and S3.

**Note S2.** Measurement system of SR-TENG and CF-TENG.

The measurement system is depicted in Fig. S2, comprising a optical platform,a linear motor, a force sensor, an adjustable slider, a support frame, and a display interface. The linear motor is used to adjust the stroke distance adopted in the test. The force sensor is utilized to quantify the driven force and to characterize the contact force between the two friction surfaces using the equation (Note S1). Its data will be displayed on the computer interface regularly. The adjustable slider has two states: the locked state (CF-TENG measurement) and the released state (SR-TENG measurement). When in the released state, the different frictional resistances can be achieved by adjusting the knob.

**References**

1. Wang Y, Liu X, Chen T, Wang H, Zhu C, Yu H, Song L, Pan X, Mi J, Lee C, Xu M. An underwater flag-like triboelectric nanogenerator for harvesting ocean current energy under extremely low velocity condition. *Nano Energy.* 2021;90:106503

2. Cho NK, Kang GY, Cho H, Jo YH, Jeong J, Shim D. Triboelectric nanogenerator integrated in a turbine using a radial rotating system and a sandwich structure. *Nano Energy.* 2023;112:108484.

3. Zhou Z, Li X, Wu Y, Zhang H, Lin Z, Meng K, Lin Z, He Q, Sun C, Yang J, et al. Wireless self-powered sensor networks driven by triboelectric nanogenerator for in-situ real time survey of environmental monitoring. *Nano Energy.* 2018;53:501-507.

4. Liu Y, Zhang D, Ji X, Xu Z, Zhang H, Mao R, Liu W, Wang J, Sun Y. Rotational contact triboelectric nanogenerator driven by water flows inspired by waterwheels and their applications for lead ion removal. *Nano Energy.* 2024;128:109800.

5. Li H, Zhang Z, Xu P, Jiang C, Yu L. A vortex-induced vibration device based on MG-TENG and research of its application in ocean current energy harvesting. *Nano Energy.* 2024;124:109457.

6. Zhou H, Wei X, Wang B, Zhang E, Wu Z, Wang ZL. A multi-layer stacked triboelectric nanogenerator based on a rotation-to-translation mechanism for fluid energy harvesting and environmental protection. *Adv. Funct. Mater.* 2023;33:2210920.

7. Yu Y, Li H, Zhao D, Gao Q, Li X, Wang J, Wang ZL, Cheng T. Material’s selection rules for high performance triboelectric nanogenerators. *Mater. Today.* 2023;64:61-71.
